# Potassium binding by carbonyl clusters, halophilic adaptation and catalysis of *Haloferax mediterranei* D-2-hydroxyacid dehydrogenase

**DOI:** 10.1038/s42003-025-08587-7

**Published:** 2025-08-06

**Authors:** Jessica Domenech, Nuttawan Pramanpol, Claudine Bisson, Sveta E. Sedelnikova, Joshua R. Barrett, Abdul A. A. B. Dakhil, Vitaliy Mykhaylyk, Ali S. Abdelhameed, Stephen E. Harding, David W. Rice, Patrick J. Baker, Juan Ferrer

**Affiliations:** 1 Dept. Bioquımica y Biología Molecular y EQA. Universidad de Alicante, Alicante, Spain; 2 School of Biosciences, University of Sheffield, Sheffield, United Kingdom; 3 Diamond Light Source, Harwell Campus, Didcot, United Kingdom; 4 National Centre for Macromolecular Hydrodynamics, School of Biosciences, University of Nottingham, College Road, Sutton Bonington, Nottingham, UK; 5 Faculty of Science and Technology, Universiti Kebangsaan Malaysia, Bangi, Selangor Malaysia; 6 Present Address: National Center for Genetic Engineering and Biotechnology, National Science and Technology Development Agency, Pathum Thani, Thailand; 7 Present Address: Evotec (UK) Ltd, Dorothy Crowfoot Hodgkin Campus 114 Innovation Drive, Milton Park, Abingdon, Oxfordshire UK; 8 Present Address: Department of Pharmaceutical Chemistry, College of Pharmacy, King Saud University, Riyadh, 11451 Kingdom of Saudi Arabia

**Keywords:** X-ray crystallography, Enzyme mechanisms, Archaeal biology, Potassium

## Abstract

Enzymes from salt-in halophiles are stable in conditions of low water activity with applications in chiral synthesis requiring organic solvents, yet the origins of such stability remains poorly understood. Here we describe the molecular basis of the reaction mechanism and dual NADH/NADPH-specificity of D2HDH, a 2-hydroxyacid dehydrogenase from the extreme halophile *Haloferax mediterranei*, an organism whose proteins have to remain active in high intracellular concentrations of KCl. Halophilic adaptations of D2HDH include the expected acidic surface and a reduction in hydrophobic surface resulting from a lower lysine content. Structure determination of crystals of D2HDH grown with KCl showed that bound K^+^ ions were coordinated predominantly by clusters of main chain protein carbonyl ligands, with no involvement of the numerous exposed surface carboxyls. Structural comparisons identified similar sites in other halophilic proteins suggesting that the generic use of carbonyl clusters to coordinate K^+^ ions may also contribute in a carboxylate-independent way to the stabilisation of the folded state of the protein in its high salt environment.

## Abstract (web summary)

Structural and biochemical studies on a halophilic D-2-hydroxyacid dehydrogenase reveal that potassium ions are bound by clusters of main chain carbonyls on the protein surface, a pattern that is shared by other halophilic proteins.

## Introduction

The natural stereoselectivity of enzymes combined with developments in expression technologies has had a significant impact on the exploitation of recombinant enzymes particularly in processes demanding chiral synthesis^1,2^. In addition, using insights provided by both structural studies and informed by random or directed evolution, new enzymes with properties of novel specificity or enhanced stability have emerged to satisfy the needs of particular synthetic applications^2,3^. However, given that our understanding of how the properties of enzymes can be rationally manipulated is still limited, such approaches remain very much in their infancy and many commercial processes rely heavily on enzymes isolated from nature^4^. In addition, in the organic solvents that are necessary to ensure substrate solubility in many chemical processes, many enzymes are unstable and show lower catalytic efficiency, potentially limiting the range of new industrial biocatalysts^5^. It has been suggested that a possible solution to this generic issue might be found in studies of enzymes from halophiles that are adapted to the dehydrating environment of high salt conditions and survive in ecological niches such as salterns, salt lakes and salt marshes^6,7^. For example, enzymes from such species that have been shown to function in organic solvent mixtures include an extracellular protease from *Halobacterium halobium*^8^, an alcohol dehydrogenase from *Haloferax volcanii*^9^, an α-amylase from *Haloarcula sp*. strain S-1^10^ and the glutamate dehydrogenase from *Halobacterium salinarum*^11^.

Halophiles belonging to the domains of *Archaea or Bacteria* that live in environments with very high salinity of up to 4–5 M NaCl (so called salt-in organisms) oppose the extracellular concentration of NaCl by accumulating similar intracellular concentrations of the osmolyte KCl. Their proteins have thus evolved to remain stable and active under these high salt and corresponding low water conditions^12,13^, unlike mesophilic proteins, which are generally inactive and with a propensity to aggregate in solutions of high ionic strength. Early sequence comparisons, biochemical studies and structural determinations on representative halophilic proteins of this type from *Haloferax mediterranei*^14^, *Haloarcula marismortumi*^13,15^, *Halobacterium salinarum*^16^ and *Salinibacter ruber*^17,18^, showed distinct differences compared to their mesophilic counterparts. The halophilic proteins had a large increase in the number of aspartate and glutamate residues, resulting in a high overall negative charge, and their surfaces were dominated by exposed carboxyl groups. In addition, a clear decrease in surface hydrophobicity was revealed, resulting from a marked reduction in the proportion of lysine residues in the halophilic sequences and the associated loss of their exposed alkyl side chains. Subsequent analyses showed that these sequence differences were genome wide^19^ acting as a fingerprint for halophilic adaptation, but with the underpinning molecular adaptive mechanisms unclear.

One proposal for the reduction in surface hydrophobicity and increase in surface acidity of halophilic proteins is to increase their stability and reduce aggregation in the high salt environment, where the hydrophobic effect is strengthened^20^. Other studies have explored the effect of the acidic surface carboxyls on halophilic adaptation by the recruitment of a tightly bound extensive water shell, allowing the protein to remain functional in its low water environment^13–15^. The precise mode of interaction of counter ions with the halophilic protein surface remains an area of considerable interest, but structural studies have yet to identify any common patterns of such interactions. Nevertheless, additional insights into halophilic adaptation provided by spectroscopic and computational methods have suggested that favourable electrostatic interactions between the acidic residues on the surface and the potassium ions in the solvent (either directly or in a water mediated arrangement) have a stabilizing effect on the folded form of the protein^21–24^, with the unfolded state destabilized because hydrated cations are preferentially excluded from the solvation shell, thereby shifting the equilibrium towards the folded form^25,26^. Taken together, these studies have suggested that the adaptations of halophilic proteins to high salt result in an optimal protein stability in their in vivo environment that is comparable to that of non-adapted mesophilic proteins in their low salinity conditions^26^, but that the relative contributions of the various molecular differences in sequence and structure to halophilic adaptation remain to be clarified.

**Fig. 1:**
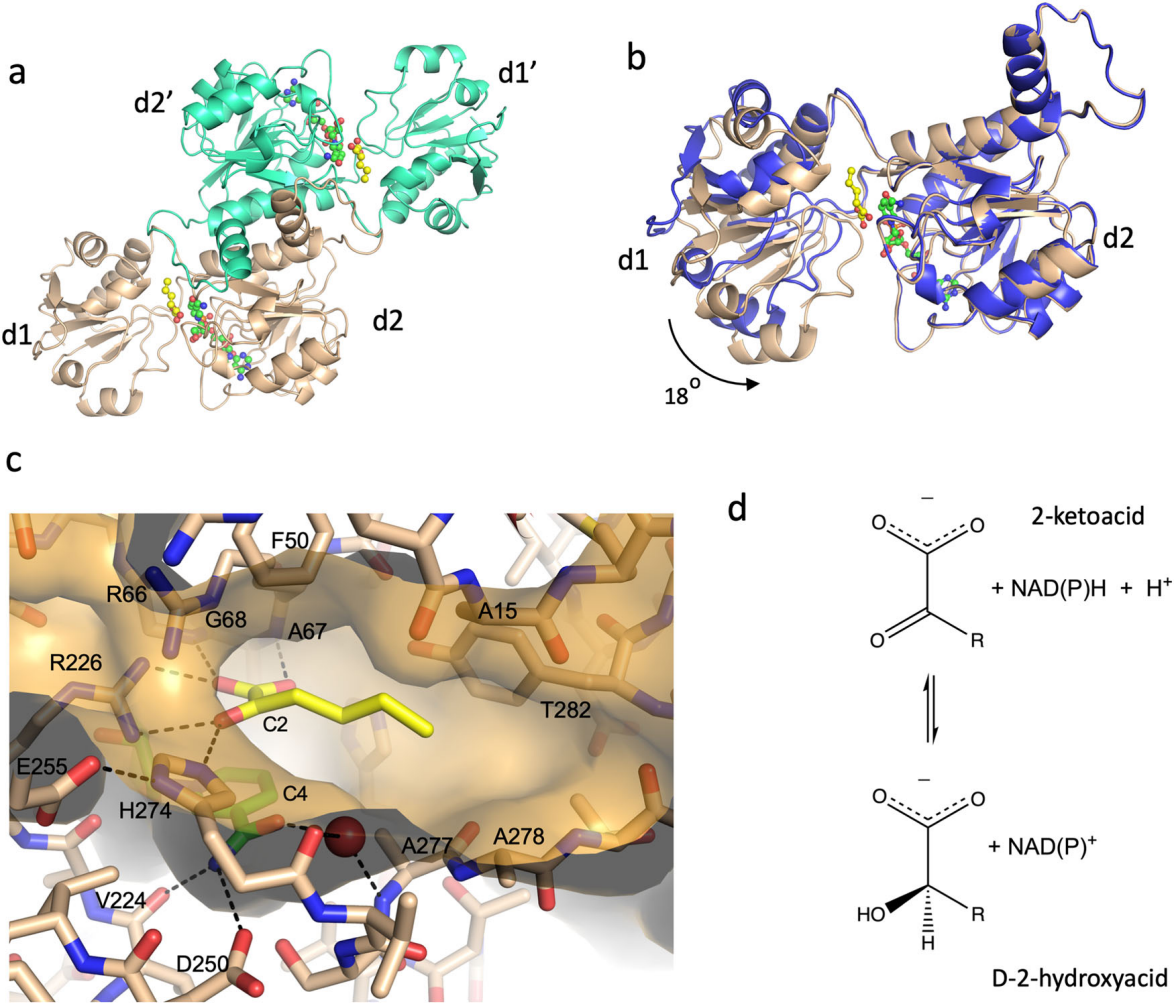
Structure of the D2HDH/NADP^+^/2-ketohexanoic acid complex (pdb:9ibe). **a** A cartoon of the D2HDH dimer (beige and teal) viewed down the molecular 2-fold with the NADP^+^ (green carbons) and 2-ketohexanoic acid (yellow carbons) in ball and stick format. **b** Monomers of the D2HDH apo enzyme (blue) and the NADP^+^/2-ketohexanoic acid complex (beige) overlapped on their d2 domains, showing domain closure on substrate binding. **c** The active site of D2HDH (beige carbons and transparent surface) showing the NADP^+^ (green carbons) and 2-ketohexanoic acid (yellow carbons), with hydrogen bonds shown as black dashes and with the NADP^+^ nicotinamide ring C4, the C2 of the substrate and interacting residues labelled. **d** The conversion of a 2-ketoacid to a D-2-hydroxyacid, the reaction catalysed by D2HDH.

In previous work, we have described the isolation, sequence and kinetic properties of a dual specificity NADH/ NADPH D-2-hydroxyacid dehydrogenase (D2HDH) from the salt-in halophile *H. mediterranei*^27^. This enzyme catalyses the NAD(P)H dependent conversion of a broad range of aliphatic 2-ketoacids to their corresponding D-2-hydroxyacids^27^ (Fig. 1). Sequence analysis has identified D2HDH as a member of the DDH clade of the D-isomer specific 2-hydroxyacid dehydrogenase enzyme family (2HADH), which has potential applications in the synthesis of chiral fine chemicals including alcohols, and hydroxy or amino acids from ketones or ketoacids^28–30^. Moreover, as D2HDH can use NADH as a cofactor, this particular dehydrogenase could be useful in reducing substrate costs in enzyme-based chemical synthesis. As *H. mediterranei* D2HDH is active in 4 M NaCl^27^ the investigation of the structure of this enzyme could facilitate its applications in chiral synthesis.

Phylogenetic analysis of the many proteins in the 2HADH family has shown that it can be subdivided into nine major subfamilies where at least one representative member has been biochemically characterised, and a further 13 much smaller groups^29^. Structural studies have shown that all members of this family share a common fold based on two domains, which cycle between open and closed conformational states that serve to close the inter-domain cleft, bringing the substrates together during catalysis^28,31^. Each domain is comprised of a similarly folded, largely parallel beta sheet flanked by alpha helices. One domain binds the dinucleotide in a similar manner to other Rossmann fold dehydrogenases^32^, and provides the major contacts between the subunits in the homodimeric structure shared by members of the 2HADH family^28,33^. Both domains provide crucial residues involved in substrate recognition. Analysis of a multisequence alignment of superfamily members shows that the active site residues that make up the strongly conserved Arg/Glu/His catalytic triad utilised by the 2HADH family are located on the surface of the nucleotide binding domain, with sequence differences in this region contributing to the diverse substrate specificity of family members^29^.

In this paper we report the results of the biochemical analysis of *H. mediterranei* D2HDH, together with the structure determination of the apo enzyme and a number of substrate complexes to provide a contribution towards an enhanced understanding of its specificity and mechanism and the features that generate salt tolerance, including the interaction of D2HDH with potassium ions.

The structures of D2HDH described in this paper have been deposited in the Protein Data Bank with the accession codes 5mh6, 5mh5, 5mhA, 8qza, 8qzb and 9ibe.

## Results

### Domain closure of D2HDH on substrate binding

The structure of the non-productive ternary complex of D2HDH with both oxidised NADP^+^ and 2-ketohexanoic acid was determined at 1.26 Å resolution, using crystals grown from protein prepared in 1 M KCl, in a crystal form with two dimers in the asymmetric unit (Table 1, pdb code:9ibe). The structure clearly shows that D2HDH shares the subunit fold and dimeric quaternary structure seen across the 2HADH family^29^, consistent with the gel filtration of the enzyme in 4 M KCl, which indicated an apparent molecular weight of 79 kDa. In addition, ultracentrifugation experiments across a range of salt concentrations showed that the dimer was always the predominant species in solution (Supplementary Fig. 1).

**Table 1:** X-ray data collection and refinement statistics for D2HDH structures

	NADP^+^ 2KHA KCl	NAD^+^ 2KHA NaCl	NADP^+^ 2KHA NaCl	NADP^+^ 2KHA 2HHA NaCl	Apo-enzyme NaCl	NAD^+^ 2KHA SO_4_^2-^ NaCl
PDB code	9ibe	8qzb	5mh5	5mha	8qza	5mh6
**Data Collection**						
Space group	P1	P1	P2_1_	P2_1_	P2_1_	P1
Cell dimensions:						
a(Å)	66.63	66.18	62.53	62.54	62.35	66.46
b(Å)	75.34	74.78	87.40	76.30	75.99	75.01
c(Å)	78.23	78.17	66.10	66.99	74.59	77.55
α(°)	108.8	109.1	90.0	90.0	90.0	109.1
β(°)	108.0	107.9	96.34	97.04	93.99	107.6
γ(°)	95.5	95.6	90.0	90.0	90.0	95.9
Subunits per asymmetric unit	4	4	2	2	2	4
Resolution (Å)^*a*^	52.28–1.26 (1.28–1.26)	46.6–1.16 (1.18–1.16)	52.51–1.4 (1.42–1.4)	38.15–1.57 (1.61–1.57)	29.4–2.25 (2.33–2.25)	46.54–1.35 (1.37–1.35)
Total reflections^*a*^	1,253,021 (60,722)	1,542,061 (62,913)	1,021,332 (50,216)	174,757 (12,974)	223,285 (6387)	577,246 (28,535)
Unique reflections^*a*^	332,819 (16,111)	427,918 (18,892)	132,820 (6288)	81,835 (5989)	32,656 (2669)	268,611 (13,112)
*R*_*pim*_^*a*^	0.059 (0.99)	0.042 (0.50)	0.036 (0.38)	0.039 (0.40)	0.041 (0.42)	0.061 (0.46)
Mean I/σ(I)^*a*^	12.0 (0.8)	17.1 (1.3)	15.4 (2.4)	13.6 (2.2)	11.9 (1.7)	23.6 (2.1)
Completeness (%)^*a*^	92.2 (89.4)	93.9 (83.7)	95.5 (91.5)	94.8 (93.7)	98.8 (88.0)	93.0 (91.7)
Multiplicity^*a*^	3.8 (3.8)	3.6 (3.3)	7.7 (8.0)	2.1 (2.2)	6.8 (5.1)	2.1 (2.2)
CC1/2^*a*^	0.99 (0.33)	0.94 (0.56)	#	#	0.99 (0.79)	#
**Refinement**	Number of non-H atoms					
Protein	9546	9590	4784	4726	4702	9534
Ligands/counter ions	269	267	145	159	7	342
Water	1575	1622	676	451	70	1768
R_work_/R_free_	0.15/0.19	0.135/0.165	0.14/0.17	0.17/0.22	0.19/0.25	0.12/0.16
Average B factor (Å^2^)						
Main chain/side chain	15.6/18.8	14.2/18.2	16.3/23.4	25.7/36.9	65.1/70.7	13.5/21.2
Ligands/counter ions	14.7	14.7	17.8	31.5	52.6	19.6
Water	28.0	30.5	27.7	32.7	44.1	33.1
Rmsd bond length (Å) / angle (°)	0.010/1.8	0.011/1.7	0.01/1.59	0.013/1.67	0.012/1.94	0.012/1.42

2KHA, 2-ketohexanoic acid; 2HHA, 2-hydroxyhexanoic acid

^a^data in brackets refers to highest resolution shell

# value not calculated

The D2HDH dimer has an overall ellipsoidal appearance in which each monomer is folded into two domains (d1; residues 1–92 and 283–308 and d2; residues 93–282). Domain d2 provides the major determinants for dimerization and dinucleotide recognition, with the nicotinamide ring of the NADP^+^ bound deep within the cleft between the two domains, adjacent to the 2-ketohexanoic acid. The other domain, d1, lies on the periphery of the dimer with the substrate making important interactions to both domains (Fig. 1a). The two domains in the subunits of both dimers adopt a closed conformation as seen in other 2HADH family members^29^, but with small differences in their relative orientations as a result of a rotation of approximately 4° about an axis between the two domains, as defined by the program DynDom^34^ with two short stretches of residues (89–92 (GIHG) and 277–282 (AATSKY) that link the two domains acting as mechanical hinges to promote interdomain motion (Supplementary Table 1).

To analyse the range of conformations that D2HDH can adopt we also determined the structure of the apo enzyme to 2.25 Å resolution (Table 1, pdb:8qza). This structure has a dimer in the asymmetric unit with clear electron density for subunit B. For subunit A, domain d2 was well defined, but the density for d1 was less good. In both subunits the two domains of the enzyme were in a more open conformation compared to those in the D2HDH/NADP^+^/2-ketohexanoic acid complex, with the cleft between them more exposed to solvent, corresponding to a rotation of approximately 18° about the same mechanical hinges (Fig. 1b, Supplementary Table 1) These differences in domain orientation in D2HDH are equivalent to the conformational changes seen in other 2HADHs^29^. In addition to the domain closure further minor conformational changes on cofactor binding involve three loops (143–146, 164–169 and 197–203) that border the adenine ring, its associated ribose group and the pyrophosphate moiety, providing residues facilitating their recognition.

### The 2-ketoacid binds in an ideal orientation for catalysis

Inspection of the electron density map of the D2HDH/NADP^+^/2-ketohexanoic acid complex (pdb:9ibe) provided clear evidence for the binding of the coenzyme and ketoacid substrates with both fitting the density unambiguously and refining with low B factors and excellent geometry (Supplementary Fig. 2a). Analysis of the interactions of the NADP^+^ with D2HDH showed that the nicotinamide ribose ring is in the C3’-endo conformation, with the nicotinamide moiety *syn* to the ribose. The orientation of the carboxamide is determined by hydrogen bond interactions between its carboxamide nitrogen and the carboxyl group of Asp250 (3.2 Å) and the main chain carbonyl group of Val224 (3.0 Å). This mode of binding is analogous to the interactions seen, for example, in D-lactate dehydrogenase (pdb:1J49)^28^ or other members of the superfamily^29^. One face of the nicotinamide ring is shielded from the solvent, packing against the side chains of a hydrophobic cluster of residues including Val95, Leu146, Val224, Ala277, such that the 4-pro-S hydrogen of the reduced nucleotide would be buried by the enzyme leaving the 4-pro-R hydrogen adjacent to the 2-ketohexanoic acid substrate and available for hydride transfer (Fig. 1c).

**Fig. 2:**
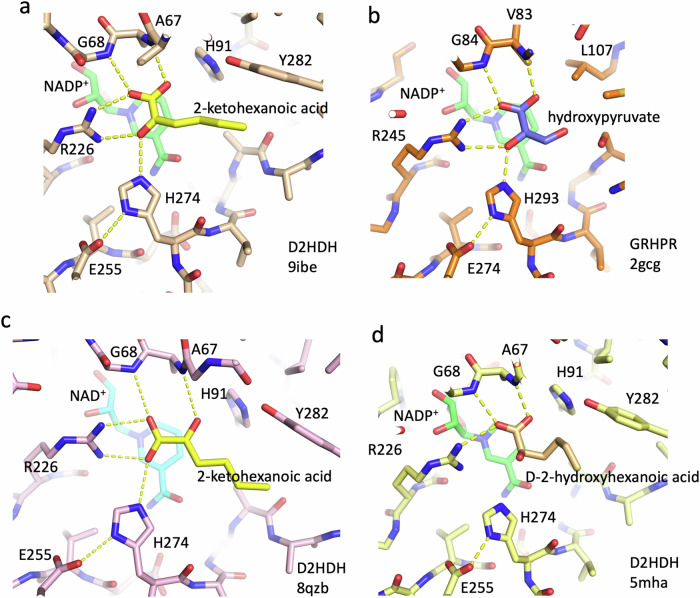
Substrate binding in the D2HDH/NAD(P)^+^/2-ketohexanoic acid complexes. **a** A close up of the binding site of 2-ketohexanoic acid (yellow carbons) in the productive orientation to D2HDH (beige carbons) in the D2HDH/NADP^+^/2-ketohexanoic acid complex (NADP^+^ green carbons, pdb:9ibe) showing close proximity of the keto moiety to the nicotinamide ring and catalytic histidine. **b** An equivalent view of the binding of hydroxypyruvate (slate carbons) to glyoxylate reductase/hydroxypyruvate reductase (GRHPR, brown carbons, pdb:2gcg), showing the close similarity in binding and active site residues to D2HDH. **c** the same view of the D2HDH/NAD^+^/2-ketohexanoic acid complex (pink carbons, NAD^+^ cyan carbons, pdb:8qzb) showing the keto acid substrate (yellow carbons) in the abortive orientation, with the keto and carboxyl moieties swapped in position compared to the productive orientation. **d** D-2-hydroxyhexanoic acid (beige carbons) bound to the active site of the D2HDH/NADP^+^ complex (light yellow and green carbons, respectively, pdb:5mha) in the productive orientation, with the C2 hydroxyl adjacent to the catalytic histidine. The active site in this complex also contains 2-ketohexanoic acid, which has been removed from the figure for clarity (Supplementary Figs. 2c and 4). Critical hydrogen bonds are highlighted (yellow dashes) with key residues and substrate atoms labelled.

The 2-ketohexanoic acid binds in a pocket adjacent to the nicotinamide ring of the NADP^+^. The substrate carboxyl oxygens (O1A and O1B) interact with the peptide NH groups of Ala 67 and Gly 68, respectively, with O1B also forming a hydrogen bond to NH2 of the guanidium group of Arg 226. The keto oxygen (O2) hydrogen bonds to NH1 of Arg 226, NH2 of Arg 66 and NE2 of the putative catalytic histidine (His 274), with its protonated NE1 also forming a hydrogen bond to the carboxyl of Glu 255. This mode of binding presents the *si* face of the substrate to the nicotinamide ring, and places the substrate C2 some 3.5 Å from C4 of the NADP^+^ nicotinamide ring, ideally positioned for hydride transfer from a reduced dinucleotide to give an alkoxide with D stereochemistry (Fig. 2a). The substrate C2 carbonyl is polarised by its interactions with His 274 and Arg 226 and lies approximately 30° out of the plane of the C1 carboxyl, decreasing the delocalisation between them, in a manner that presumably lowers the energy barrier for the conversion of the C2 carbon from *sp2* to *sp3* as hydride transfer occurs. To complete the reaction the histidine donates a proton to the alkoxide to produce the D-hydroxy acid product (Supplementary Fig. 3). This proposed mechanism and the mode of substrate binding are highly reminiscent of those derived from the structures of ketoacid complexes of the enzymes in the wider 2HADH family^29,35,36^. In particular, the four highly conserved active site residues identified across the family (a glycine, an arginine, a glutamate and a histidine) are equivalent to Gly 67, Arg 226, Glu 255 and His 274 in D2HDH (Fig. 2b).

### Dual coenzyme specificity of D2HDH and its activity in organic solvents

**Fig. 3:**
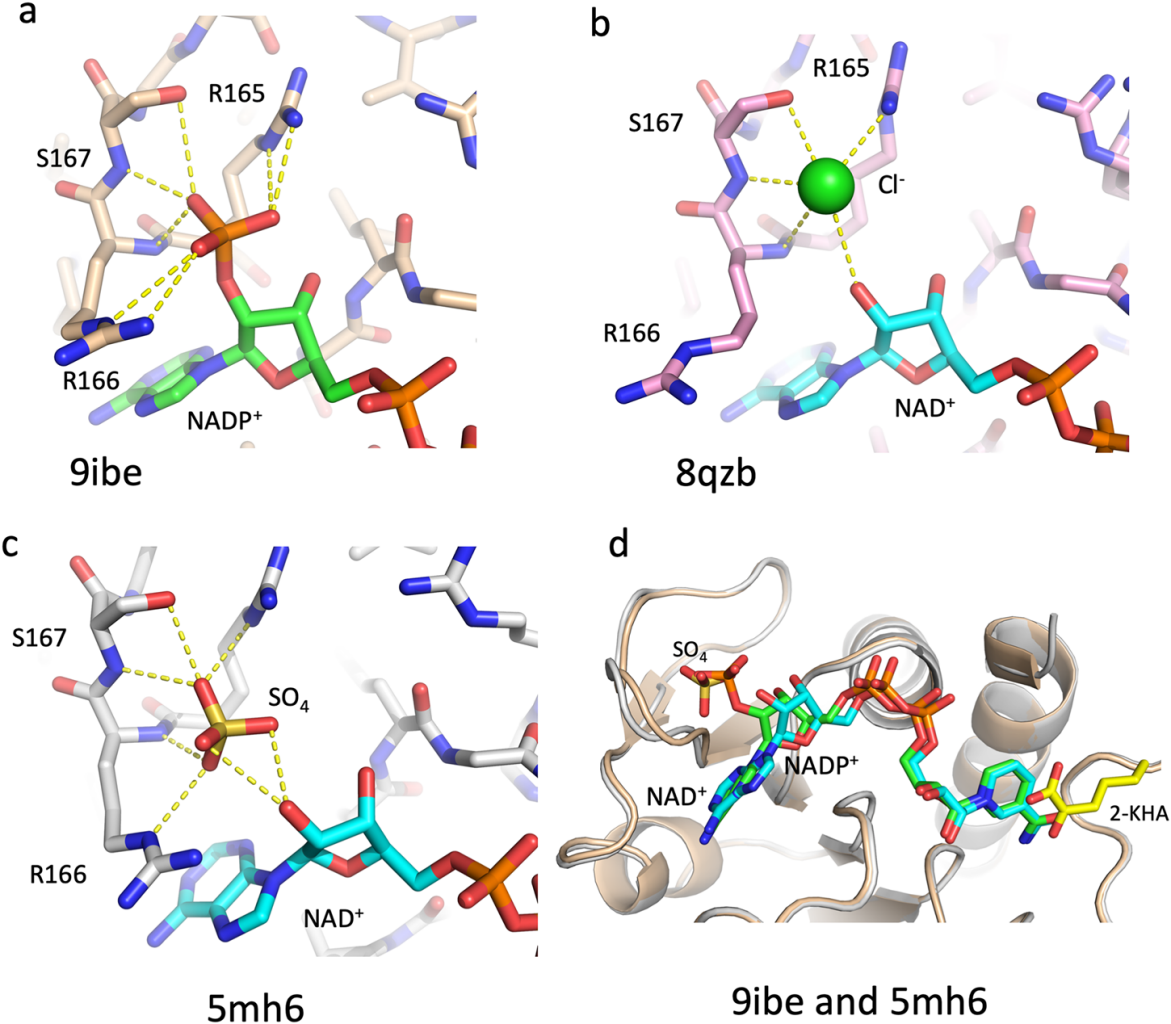
Cofactor adenine ribose interactions. **a** A close up of the binding site of the adenine ribose ring in the NADP^+^/D2HDH/2-ketohexanoic acid complex (beige carbons, pdb:9ibe) showing the position of the cofactor (green) the 2’ phosphate, and interactions to the protein (yellow dashes). **b** The same view for the NAD^+^/D2HDH/2-ketohexanoic acid complex (pink carbons, cyan NADP^+^, pdb:8qzb), showing the chloride ion (green sphere) binding in the equivalent position to the 2’ phosphate of NADP^+^. **c** Equivalent view of the NAD^+^/D2HDH/2-ketohexanoic acid/sulfate complex (white carbons, pdb:5mh6) showing the associated sulfate ion (yellow) binding in a similar position to the Cl^-^ ion. **d** A cartoon representation of the superimposed co-factor binding domains of the NAD^+^/SO_4_ and NADP^+^/2-keto acid complexes (coloured as above) highlighting differences in position of the sulfate compared to the ribose phosphate and illustrating the movement of the wing of this domain (Gly 163 - Ala 190) to accommodate the sulfate in the NAD^+^/SO_4_ complex. In all the complexes the positioning the co-factor nicotinamide ring and 2-keto acid substrate (2-KHA, yellow) is essentially identical.

In the D2HDH/NADP^+^/2-ketohexanoic acid complex (pdb:9ibe) the adenine ring of NADP^+^ sits in a mainly hydrophobic pocket bounded by residues Val141 Gly142, Val164, Pro180, Leu183 Thr197, Pro198 and Met 206. The adenine ribose ring adopts a C2’-endo conformation with its 3’-hydroxyl interacting with the main chain NH of Leu 143. The 2’ phosphate moiety interacts with the side chains of Arg 165 and Ser 167, and the main chain NHs of both Arg 166 and Ser 167 and also in two subunits (A and D) to the side chain of Arg 166 (Fig. 3a). In subunits B and C Arg 166 makes interactions to symmetry related molecules in the crystal lattice, rather than to the 2’ phosphate. The pyrophosphate group of the dinucleotide lies at the N-terminal end of helix α6, where it is stabilised by the helix dipole and hydrogen bonds to main-chain nitrogen atoms in the first turn of the helix (Thr 145 and Leu1 46), as seen in all other Rossmann fold enzymes^37^.

To explore the ability of this enzyme to use both NADP^+^ and NAD^+^ as cofactors in the reaction the structure of a D2HDH/NAD^+^/2-ketohexanoic acid complex was also determined at 1.16 Å resolution, from crystals grown from protein prepared in 2 M NaCl (Table 1, pdb:8qzb). Both subunits of the two dimers of the asymmetric unit were again in a closed conformation, but with small differences in the extent of domain closure (Supplementary Table 1). Although the NAD^+^ in this complex (pdb:8qzb) was bound similarly to the NADP^+^ described above, the 2’ OH of the adenine ribose ring of NAD^+^ interacted with the main chain NH of Arg 166 and lay 3.5 Å from a chloride ion (identified by its anomalous scattering signal). This chloride lies in approximately the same position as the 2’ phosphate of the NADP^+^ and interacts with the guanidyl group of Arg165 and the main chain NH and side chain oxygen of Ser 167 (Fig. 3b). Comparing the two structures movements of up to 1.5 Å in the residues of the Arg 165-Val 171 loop, that lies adjacent to the 2’ hydroxyl or 2’ phosphate groups of the two nucleotides, occur (Fig. 3d). The additional interactions of the 2’ phosphate with the enzyme presumably contribute to the stronger binding of NADPH compared to NADH when assayed under conditions of either 2 M NaCl^27^ or 4 M KCl (Table 2).

**Table 2:** Kinetic Parameters of D2HDH in 4 M KCl for NADPH and NADH using 2-ketohexanoic acid as substrate and for 2-ketohexanoic acid using NADPH or NADH as cofactor

	K_M_ (mM)	V_max_ (U/mg)	K_CAT_ (min^-1^)	K_CAT_/K_M_ (mM^-1^ min^-1^)
NADPH (2-ketohexanoic acid as substrate)	0.014 ± 0.004	2.5 ± 0.1	83 ± 4	5928
NADH (2-ketohexanoic acid as substrate)	0.097 ± 0.018	4.4 ± 0.3	149 ± 10	1536
2-ketohexanoic acid (NADPH as cofactor)	0.46 ± 0.03	2.5 ± 0.05	83 ± 2	180
2-ketohexanoic acid (NADH as cofactor)	1.48 ± 0.16	3.8 ± 0.1	124 ± 4	84

Assays were performed in 4 M KCl, 50 mM HEPES pH 8.0 with 0.05 - 20 mM 2-ketohexanoic acid 0.25 mM NADH; 0.05 - 4 mM 2-ketohexanoic acid, 0.25 mM NADPH; 0.025 - 0.3 mM NADPH, 4 mM 2-ketohexanoic acid or 0.05 - 0.3 mM NADH, 20 mM 2-ketohexanoic acid. K_M_ and V_max_ were calculated using http://www.ic50.tk/kmvmax.html

**Fig. 4:**
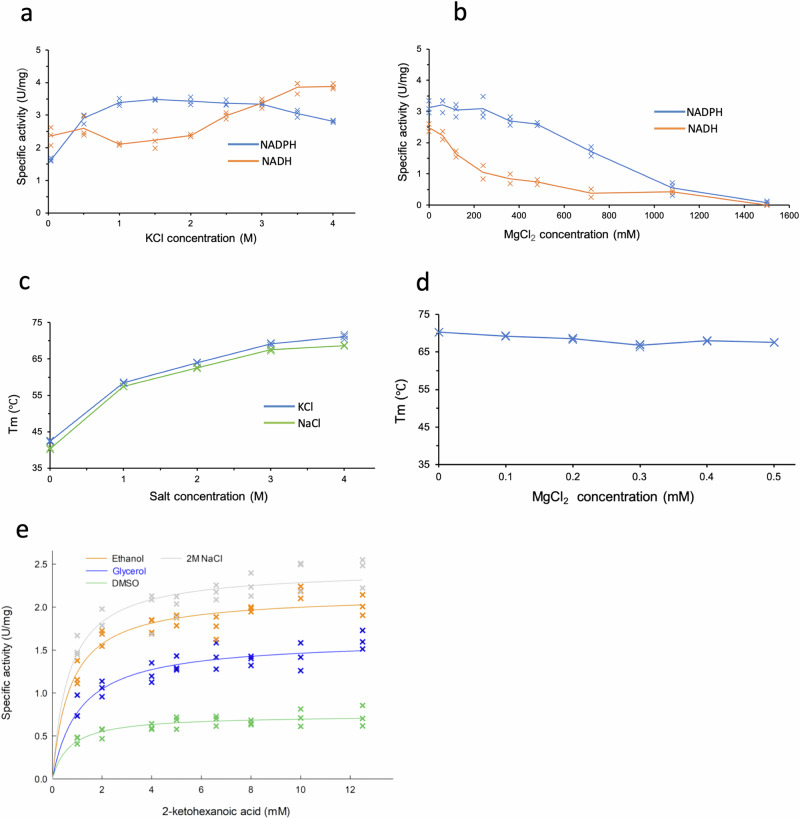
D2HDH Activity and stability assays. The specific activity of D2HDH as a function of **a** KCl concentration and **b** MgCl_2_ concentration in 1 M KCl, with 2-ketohexanoic acid as the substrate and NADPH (blue) or NADH (orange) as cofactor. **c** The thermostability (Tm) of D2HDH as a function of KCl (blue) or NaCl (green) concentration and **d** D2HDH thermostability in 3 M KCl varying MgCl_2_ concentration. (e) The specific activity of D2HDH in 2 M NaCl (grey) and also with 20% of DMSO (green), glycerol (blue) and ethanol (orange) as a function of 2-ketohexanoic acid concentration. For each graph (a-e) data points (n = 3, except (b) NADH, n = 2) are shown as crosses with the solid line the average for (a-d) and for (e) a Michalis-Menten fitted curve.

To investigate the impact of chloride binding on D2HDH activity we examined the effect of varying concentrations of KCl on the enzyme activity with both NADH and NADPH, which showed different activity profiles for the two cofactors. For NADPH, the activity increases as KCl rises to plateau at 1 M with a 20% loss of activity at KCl concentrations above 3 M (Fig. 4a). Given the presence of a chloride ion in the structure of the D2HDH/NAD^+^/-2-ketohexanoic acid complex at the 2’ phosphate binding site of NADP^+^, the decrease in the NADPH-dependent activity of the enzyme at high concentrations of KCl was consistent with the Cl^-^ ion competing with the 2’ phosphate for the same binding site on the protein. In contrast, for assays with NADH as the cofactor there is a gradual increase in D2HDH activity as KCl concentration increases, to a level (at 4 M KCl) equivalent to the maximum activity with NADPH (Fig. 4a). Compared to the loss in activity seen with NADPH at high KCl concentrations, the absence of any similar reduction with NADH is consistent with the independent binding sites for the chloride ion and the 2’ ribose hydroxyl of NADH (Fig. 4b). At low KCl concentrations the predominant species would be a NADH/enzyme complex, but as the KCl concentration rises, the binding of Cl^-^ at the 2’ phosphate site would increase, to give a NADH/Cl^-^ D2HDH complex which would mimic that of the NADPH enzyme.

A separate structure of D2HDH with NAD^+^ and 2-ketohexanoic acid was also determined at 1.35 Å resolution, using protein also prepared in 2 M NaCl, but using a modified purification protocol in which the gel filtration step had been omitted leaving residual sulfate in the protein preparation for crystallisation (Table 1, pdb: 5mh6). In this structure, a sulfate ion that co-purified with the protein occupied the approximate position of the chloride ion seen in the equivalent NAD^+^ complex prepared using enzyme with the additional gel filtration step (pdb:8qzb), or the 2’ phosphate of the NADP^+^ complex (pdb:9ibe). As this sulfate mimics the 2’ phosphate of NADP^+^, but in a non-covalently linked manner, both the sulfate, the adenine ring of the NAD^+^ and its associated ribose are shifted by up to 2.0 Å compared to their equivalents in the NADP^+^ complex (Fig. 3c). Similar, though not identical movements of the 165-171 loop and in particular the side chains of Arg 165 and Arg 166 that accompany the recognition of the NADP^+^ adenine ribose phosphate again occur. In the structure with NADP^+^ (pdb:9ibe) the C5-O5 bond of the NADP^+^ adenine ribose adopts a staggered conformation (average torsion angle 170°), whereas in the structure with NAD^+^ and sulfate (pdb:5mh6), the equivalent bond rotates by 20° and is partially eclipsed (average torsion angle 151°). In the structure with NAD^+^ and Cl^-^ (pdb:8qzb) the C5-O5 bond is less eclipsed and more like that seen in the NADP^+^ structure (average torsion angle 164°). These differences presumably reflect the subtly different compromises in binding that arise as a result of the mimicking of NADP^+^ by NAD^+^ with sulfate or chloride. Nevertheless, in all three of these structures the nicotinamide ring of the cofactor and the position of the substrate remain in essentially the same position (Fig. 3d).

As the family of D-2-hydroxyacid dehydrogenases have the potential to be exploited in biotechnology^27^, we examined the determinants of substrate specificity. This showed that the 2-ketohexanoic acid binds in a pocket adjacent to the nicotinamide ring of the NADP^+^, with its alkyl chain in a staggered conformation and bounded by Ala 15, Phe 50, Arg 66, Ala 277, Ala 278, and Tyr 282 (Fig. 1c). The broad shape of this largely hydrophobic pocket provides an explanation for the substrate promiscuity of D2HDH. Since D2HDH is isolated from a halophile, we further explored the activity of the enzyme in different organic solvents, often an essential property for industrial biotransformations^5^. Assays showed that the enzyme retained between 25–80% activity in the presence of DMSO, glycerol or ethanol, additives that could be used to improve the solubility of hydrophobic substrates (Fig. 4e). This finding, together with the ability of D2HDH to accept numerous 2-ketoacid substrates^27^ and to use the less expensive NADH cofactor at high chloride ion concentrations suggests the enzyme might be a suitable vehicle for the production of a range of chirally pure D-2-hydroxyacids.

### The 2-ketoacid can bind to the active site in productive and abortive orientations

Inspection of the electron density map of both non-productive ternary complexes of D2HDH with 2-ketohexanoic acid and either NAD^+^ and chloride or NAD^+^ and sulphate (pdbs:8qzb; 5mh6), provided clear evidence for the binding of the ketoacid at the active site, adjacent to the nicotinamide ring. Unexpectedly, the substrate bound in a quite different orientation, with the carboxylate and keto groups planar and with their oxygens hydrogen bonded to NH1 and NH2 of Arg 226, and the main chain NH of Ala67, respectively (Fig. 2c, Supplementary Fig. 2b). Compared to the productive mode of binding of the substrate in the D2HDH/NADP^+^/2-ketohexanoic acid complex described above (pdb:9ibe, Fig. 2a) this effectively exchanges the positions of O1A and the keto oxygen O2, presenting the *re* face, rather than the *si* face, of the substrate towards the nicotinamide ring. This alternative mode of substrate recognition is catalytically abortive as the carbonyl is no longer adjacent to the acid/base catalyst, His 274, with no other residue able to provide a similar function. These two distinct modes of substrate binding in the active site of D2HDH (productive and abortive) are possible as the three substrate oxygen atoms recognised by the enzyme are related by an approximate 2-fold axis of rotation in the plane of the ketoacid, a pseudosymmetry shared by all 2-ketoacids (Fig. 2). In both the productive and abortive modes of substrate binding the 2-ketohexanoic acid alkyl side chain occupies a similar position in the active site, but is twisted as a result of a 120° rotation to the C2-C3 torsion angle. To confirm that this different mode of substrate binding was not due to the incorporation of a different cofactor in the respective complexes (NAD^+^ or NADP^+^) a further structure of D2HDH in complex with NADP^+^/2-ketohexanoic acid was determined using NaCl prepared protein (Table 1, pdb:5mh5). In this structure the ketoacid was also bound in the abortive orientation, showing that the difference was independent of the nature of the co-factor.

### Characterization of hydroxy acid binding

Thus far, to our knowledge, no structures of the wider DDH family have been determined with a reduced hydroxy acid substrate^29^. To better understand the mode of product recognition by D2HDH, we attempted to grow crystals of a non-productive ternary complex of the enzyme with a variety of 2-hydroxyacids (including 2-hydroxyhexanoic acid) and NAD(P)H, to complement the 2-ketoacid structures described above, but the crystals that were obtained were of poor quality, and not suitable for structure determination. However, crystals grown in the presence of 5 mM NADPH and 50 mM 2-ketohexanoic acid were suitable for structural analysis (Table 1, pdb:5mha). Under these crystallization conditions the enzyme would be expected to turn over the substrates leading to crystals that might contain a mixture of oxidised and reduced components. Given that the equilibrium constant favours the production of the reduced substrate^38^, reaction turnover would lead to significant oxidation of the NAD(P)H to NAD(P)^+^ and the concomitant accumulation of mM concentrations of D-2-hydroxyhexanoic acid, albeit in a mixture with unreacted ketoacid in excess compared to the protein concentration (~0.1 mM). In this structure the refined electron density was planar for the nicotinamide ring consistent with the major component bound at the active site being the oxidised cofactor^39,40^. Although the electron density for the substrate was clear, it could not be explained by either a ketoacid or a D-2-hydroxyacid alone (Supplementary Fig. 2c). For example, fitting the density with a ketoacid left a significant additional bulge close to the substrate C2, particularly for subunit B (Supplementary Fig. 4). This suggested that a mixture of reduced and oxidised substrate was present. Careful refinement of this structure showed that the substrate density could be best explained as a 2:1 mixture of 2-ketohexanoic acid in the abortive orientation and D-2-hydroxyhexanoic acid in a productive orientation. Consistent with the mechanism proposed earlier (Supplementary Fig. 3), the C2 hydroxyl of the D-2-hydroxy acid is positioned 4.0 Å away from NE2 of the putative catalytic histidine (His 274), but requiring a further conformation change to the reduce the distance between these atoms to allow catalysis to proceed (Fig. 2d). We presume that turnover in the crystal of the productive complex between the oxidised cofactor and the reduced substrate has been prevented by crystal contacts.

### D2HDH shows the classic features of a halophilic enzyme

Analysis of the structure confirmed that, as expected, D2HDH has the characteristic halophilic adaptation of a surface dominated by a large number of carboxyl groups and is an outlier compared to mesophilic homologues from the wider 2HADH family (Supplementary Fig. 5b, Supplementary Table 2). The distribution of the acidic residues across the surface of D2HDH is non-uniform, with one face of the D2HDH dimer less acidic than the rest of the molecule and with regions involved in substrate and cofactor recognition, and subunit assembly largely devoid of acidic residues. D2HDH also shows a loss of hydrophobic surface arising from reduced lysine content as seen in other halophilic proteins. An increase in the proportion of threonine and a decrease in phenylalanine residues has been suggested as a halophilic adaptation in other halophilic proteins^41,42^, but is not seen in D2HDH (Supplementary Table 2). These proposed halophilic adaptations in D2HDH are consistent with the global differences in amino acid composition of halophilic proteins implied by analysis of salt in halophile genomes^19^, with comparisons reported previously^13–16^ and with a wider analysis we have conducted as part of this work on ten proteins from salt-in halophiles whose structures have been determined at a resolution greater than 1.6 Å (Supplementary Data 1).

As the organisation of the water structure and the presence of counter ions surrounding the acidic protein surface has been implicated in the folding and stability of halophilic proteins^43^, we analysed the water structure of D2HDH using the model derived from the highest resolution data (the 1.16 Å structure of the D2HDH/NAD^+^/2-ketohexanoic acid complex, pdb:8qzb). D2HDH has an extensive solvation shell with many water molecules located in depressions in the enzyme surface and forming extensive networks with each other and the protein (Supplementary Fig. 5c). We noted that in general there were fewer waters surrounding the peripheral domains (d1) of the D2HDH subunits compared to the dimerization domains (d2) (Supplementary Fig. 5c). In part, this is likely due the greater mobility of domain 1, which is reflected in the higher overall temperature factors for this domain (d1 average 24 Å^2^, d2 average 11 Å^2^, Supplementary Table 3). Nevertheless, the average of 1.3 waters per residue is in line with that seen for other non-halophilic protein structures determined at a similar resolution and is thus unlikely to be solely due to the halophilic nature of D2HDH. In the analysis of solvent structure around the surface of mesophilic proteins, pentagonal arrays of waters adjacent to solvent-exposed hydrophobic residues have been seen, as for example in the seed storage protein crambin^44^, the non-halophilic *Chloroflexus aurantiacus* MalDH homologue^45^ and also in the structure of halophilic *H. mediterranei* glucose dehydrogenase^14^. However, no pentagonal arrays of water molecules could be identified in D2HDH, suggesting that such arrays are not a feature of halophilic adaptation in this enzyme.

### K^+^ ions bind to clusters of carbonyls in D2HDH

The crystallization of D2HDH in the presence of high concentrations of KCl has allowed us to identify binding sites of these counter ions to investigate their role in halophilic adaptation. The structure of the D2HDH/NADP^+^/2-ketohexanoic acid complex (pdb:9ibe) contained 23 K^+^ ions that were unambiguously confirmed by a combination of their environments, coordination chemistry, ligand distances and anomalous scattering data collected at energies above and below the potassium absorption edge. Similarly, 5 Cl^-^ and 12 Mg^2+^ ions were also identified.

**Fig. 5:**
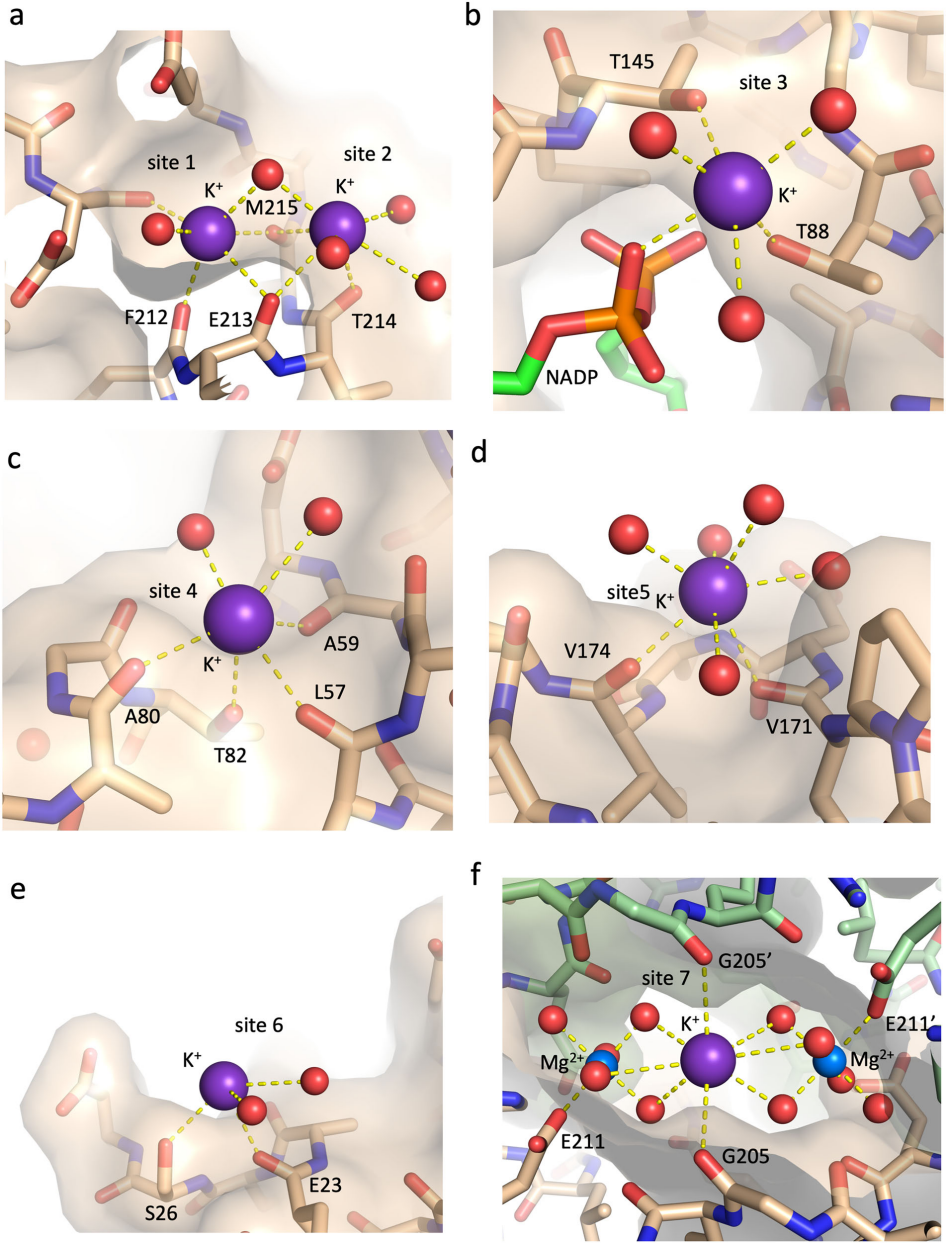
D2HDH uses carbonyl and hydroxyl groups to bind potassium ions. The seven K^+^ ion binding sites (**a–f**) in the D2HDH/NADP^+^/2-ketohexanoic complex (beige carbons and transparent surface pdb:9ibe), with K^+^ (purple), Mg^2+^ (blue) and waters (red) highlighted as spheres and with the protein ligands labelled. Note the preponderance of protein carbonyl ligands to the K^+^ ions. **f** shows site 7 that lies between two symmetry related D2HDH monomers in the crystal (beige and green), with the potassium ion bridging two intermolecular Mg^2+^ ions.

Seven distinct potassium ion binding sites (sites 1–7, Fig. 5, Supplementary Table 4) could be identified in the D2HDH monomer, predominantly lying on the less acidic face of the dimer (Supplementary Fig. 5a). In sites 1–5 potassium ions were bound to each of the four subunits of the two dimers in the asymmetric unit, except for site 5, where the close approach of a crystal packing subunit precluded the site formation in subunit D. The K^+^ ions in sites 1 and 2 are separated by 3.9 Å, and share the main chain carbonyls of Glu 213 and Met 215 together with a water molecule as bridging ligands (Fig. 5a). Site 6 was found in only one subunit of each dimer (subunits A and C). Site 7 occurred between adjacent dimers in the crystal lattice, with two such sites present in the asymmetric unit. In this site a K^+^ ion lies between two symmetry-related hydrated Mg^2+^ ions, with the K^+^ ion coordinating three waters bound to each of the Mg^2+^ ions, together with the carbonyl oxygen of Gly 205 and its symmetry-related mate (Fig. 5f).

In sites 1–6 the principal protein ligands to the K^+^ ions were provided by clusters of surface exposed main chain carbonyl oxygens, with no involvement of any glutamate or aspartate carboxyl groups (Fig. 5 and Supplementary Table 4). Three sites involve additional interactions from the oxygens of serine or threonine side chain hydroxyls or the NADP^+^ pyrophosphate. Water molecules completed the K^+^ coordination sphere to give coordination numbers in all the sites between six and eight, except in site 6, where coordinating waters were not visible in the electron density map.

Whilst it has been suggested that the formation of protein crystals might exclude counter ions associated with the protein surface and its solvation shell^45^, the finding that the potassium ions bound in sites 1–5 are common to all subunits, not involved crystal contacts and were coordinated by carbonyl or hydroxyl ligands, rather than the numerous negatively charged carboxyl groups, suggests that these cations represent a class of strongly bound ions that may contribute to halophilic adaptation. This is supported by the increase in activity of D2HDH as the K^+^ concentration increases (Fig. 4a). In addition, experiments varying the K^+^ concentration on the stability of D2HDH showed that its melting temperature (Tm) increased by ~30 °C as the KCl concentration was raised from 0.05 to 4 M (Fig. 4c), possibly suggesting that potassium binding has a stabilising effect on the enzyme. However, caveats to the interpretation of changes in Tm are that it measures differences in the thermal resistance of the protein to unfolding under the tested conditions rather than measuring differences in thermodynamic stability (the free energy difference between the folded and unfolded states) and that irreversible aggregation at high temperature precludes measurement of the whole stability curve^46^. Nevertheless, the measurements of Tm clearly indicate that the structure and stability of the enzyme are influenced by the increasing concentration of potassium, in a way that mirrors the increase in activity as potassium concentration increases.

As D2HDH is also very active in the presence of high concentrations of Na^+^ and shows a similar increase in Tm with increasing NaCl concentration (Fig. 4c), we compared the positions of Na^+^ ions in the structure of the D2HDH/ NAD^+^/2-ketohexanoic acid complex (pdb:8qzb) whose crystals were grown in the presence of NaCl, to the K^+^ sites discussed above. Three well defined Na^+^ binding sites per subunit could be confidently assigned, two of which were in common with sites 1 and 3 in the K^+^ structure. This is possible because the protein ligands in these two sites are clustered together in such a way as to permit the Na^+^ ion to move closer to them to accommodate its smaller ionic radius, whilst allowing associated differences in the water structure (Supplementary Fig. 6).

Although numerous bound K^+^ ions were present in the D2HDH/NADP^+^/2-ketohexanoic acid complex (pdb:9ibe), only one Cl^-^ ion, lying at the N-terminal end of a helix adjacent to the main chain nitrogen of Glu 232, was bound to each subunit. A further Cl^-^ ion was found close to the guanidinyl group of Arg20 in one subunit (A) in a region of crystal contacts. These Cl^-^ sites are distinct from the additional Cl^-^ ion that binds adjacent to the 2’ adenine ribose hydroxyl in the NAD^+^ complexes.

### Magnesium ions bind at crystal contacts

A possible role for Mg^2+^ ions in halophilic adaptation has been proposed for other proteins^43^, and in addition to the bound K^+^ and Cl^-^ ions in the D2HDH/NADP^+^/2-ketohexanoic acid complex (pdb:9ibe) the structure also contained Mg^2+^ ions as the protein was crystallised from solutions containing magnesium acetate. The 12 Mg^2+^ ions in the asymmetric unit have octahedral coordination with the cations making either direct (nine instances) or water-mediated (three instances) interactions to a surface carboxyl group, consistent with the high charge density for Mg^2+^^47^. In contrast to the K^+^ ligands, none of the Mg^2+^ ligands were provided by protein carbonyl or hydroxyl groups and importantly, all of these Mg^2+^ ions were located at or near regions of crystal contacts, with one or more of their coordinating water ligands forming direct or water mediated interactions to residues of a neighbouring subunit, most frequently a surface carboxyl (Fig. 5f and Supplementary Fig. 6). Assays in the presence of 1 M KCl using either NADPH or NADH as the cofactor showed the activity of D2HDH fell off substantially as MgCl_2_ concentration increased, to approximately zero at 1.5 M MgCl_2_ (Fig. 4b). Similar loss of activity with high MgCl_2_ has been seen in MalDH from both *H. marismortui* and *S. ruber*^48^ and has been attributed to protein destabilization at high MgCl_2_ concentrations^22,49^. In addition, varying the Mg^2+^ concentration between 0–0.5 M in the presence 3 M KCl showed no increase in the Tm of D2HDH (Fig. 4d), similar to proteome-wide observations on a range of halophilic species^50^. Together with the Mg^2+^ binding sites lying exclusively at crystal packing interfaces, this supports the view that in *H. mediterranei* D2HDH Mg^2+^ ions play little additional role in halophilic adaptation.

## Discussion

The structural studies described above have provided a mechanism for D2HDH and contributed to a more complete understanding of the structure-function relationships of the wider 2HADH family by providing a structure for the mode of binding of both the 2-ketoacid and the D-2-hydroxyacid. Moreover, these studies have provided an explanation for the dual cofactor specificity of D2HDH, the molecular basis of the tighter binding of the enzyme for NADPH compared to NADH and its broad substrate specificity. Interestingly, the structures of the D2HDH complexes determined in this study have shown that, dependent upon the crystallisation conditions used, the 2-ketoacid substrate can be trapped in the crystal structure in two different orientations, representing a catalytic or abortive mode of binding. This situation is possible as a result of the pseudo symmetry in the position of the three oxygen atoms of a 2-ketoacid, which allows for two distinct modes of substrate recognition, each using interactions with the same set of functional groups in the active site. A similar problem could potentially occur in studies of other 2-hydroxyacid dehydrogenases given the equivalent pseudo symmetry in their substrates and our study suggests that considerable care needs to be exercised in deriving conclusions from similar structural studies.

The use of anomalous scattering to identify counter ion binding sites in D2HDH that might also be found in vivo has only identified a very limited number of Cl^-^ ions, with no consistent pattern of interaction such that their role in halophilic adaptation is not clear. However, multiple K^+^ ions were identified with strong similarities in the manner in which they bind to the protein. Significantly, the coordination of the K^+^ ions does not involve any of the plethora of surface carboxyl groups, but rather is associated with binding sites formed mainly from clusters of main-chain carbonyl groups. Specifically, of the 65 protein ligands to the 23 K^+^ ions in the asymmetric unit of the D2HDH structure (2 protein dimers), 52 are carbonyl oxygens and 13 are hydroxyls. The architecture of these K^+^ binding sites arises because carbonyl and hydroxyl groups frequently occur close in space to each other on the protein surface, such as in helical turns, in geometric arrangements consistent with that required for such a site and which can also link and potentially stabilise different regions of the polypeptide chain together (Fig. 5).

To examine whether the pattern of K^+^ ion coordination by carbonyl clusters in D2HDH is also present in other proteins from salt-in-halophiles, we identified four other halophilic proteins crystallised in the presence of KCl in the PDB that included bound potassium ions (excluding structures of the ribosome and members of the rhodopsin family). This analysis showed that carbonyl oxygens are the predominant K^+^ ligands in *H. mediterranei* glucose dehydrogenase (GlcDH, pdb:2b5w), where of the 5 K^+^ ions in the asymmetric unit (one subunit of a dimer), 8 of the protein ligands are carbonyl oxygens, with one hydroxyl and only a single carboxylate from an aspartate residue^14^. Equally, in the highest resolution structure of *H. volcanii* malate synthase (MSH, pdb:3oyz), the predominant potassium ligands are carbonyl groups (11 protein ligands to the 3 K^+^ ions associated with each monomer: eight main chain carbonyls, two amide oxygens, two hydroxyl groups and a single carboxyl), with one of the K^+^ ions lying at the trimer interface and linked through a carbonyl to the adjacent subunit^51^.

In *H. marismortui* malate dehydrogenase (hMDH, pdb:7q3x, unpublished) and *H. marismortui* ferredoxin (pdb:1doi)^23,52^ the pattern of K^+^ ligation again involves many carbonyl groups, but with more carboxyl ligands being involved. In hMDH there are 57 protein ligands to the 25 K^+^ ions in the asymmetric unit (a hMDH tetramer) of which 36 are carbonyl oxygens, but with 21 of the ligands being carboxyl oxygens (17 aspartate and 4 glutamate residues). A similar situation occurs in *H. marismortui* ferredoxin, where of the 17 protein ligands to the 6 K^+^ ions in the asymmetric unit (a ferredoxin monomer) 10 ligands are carbonyl oxygens, 1 is a threonine hydroxyl and 6 are carboxyl oxygens (5 aspartate and 1 glutamate residues).

However, considering only the intra-subunit K^+^ sites in both of these proteins (11 and 4 sites in hMDH and ferredoxin, respectively) the majority of the protein K^+^ ligands are carbonyl oxygens (21/25 in hMDH and 7/10 in ferredoxin), with just two aspartate carboxyls in hMDH and one aspartate and one glutamate carboxyl in ferredoxin involved, very similar to the situations seen in *H. mediterranei* D2HDH and GlcDH and *H. volcanii* MSH. In contrast, all the K^+^ sites with carboxyl ligands in ferredoxin occur at crystal contacts between protein chains and hence are likely to be artefacts of crystallisation. This is not solely the case in hMDH, where many of K^+^ ion sites with carboxyl ligands occur at subunit interfaces of the tetramer. Using carboxyl groups in this manner may arise in part because it is more difficult to arrange groups of carbonyls on two opposing surfaces to bind a K^+^ ion, whereas the flexibility and solvent exposed side chains of aspartate and glutamate can facilitate the design of such inter-subunit K^+^ sites, albeit with an apparent preference for the use of aspartate, rather than glutamate, carboxyl groups. Furthermore, whilst the potassium ions found at the subunit interfaces in hMDH may add to the stability of the oligomer, there appears to be no general requirement for K^+^ ions in such interfaces in halophilic proteins as the dimer interfaces in both D2HDH and GlcDH do not use linking cations.

Studies have shown that halophilic protein adaptation to high salt arises from a combination of stabilisation of the folded state of the halophilic protein, together with a destabilisation of the unfolded state^25^. The analysis presented here of the structure of the folded state of D2HDH has again highlighted the importance of the accepted nature of the acidic surface to halophilicity, and the universal reduction in surface lysine residues in such proteins and the consequent reduction in hydrophobic surface^14,16,20^. Equally, the absence of significant arrays of pentagonally or hexagonally arranged water molecules in the solvation shell of D2HDH is consistent with their proposed disruption by the extensive acidic surface of halophilic proteins compared to their non-halophilic homologues^45^.

A further contribution to the stabilisation of halophilic proteins arises from electrostatic interactions between the protein surface and solvent cations, with many studies focussing on the importance of interactions between K^+^ ions and the abundant surface carboxylates^21–26^. In D2HDH the disposition of the surface carboxyls, particularly those of the longer glutamate side chains, are remote from other potential oxygen atom K^+^ ligands, suggesting that any binding of K^+^ ions to carboxylates in D2HDH may well be weak, transient and difficult to observe in a crystallographic experiment. However, protein-bound K^+^ ions can be clearly identified in the structure of D2HDH, but are coordinated primarily by clusters of surface main chain carbonyl groups, rather than involving side chain carboxyls. The presence of carbonyl groups in the coordination shell of bound potassium ions have been previously reported for *H. marismortui* ferredoxin^52^ and *H. mediterranei* GlcDH^14^, but in both cases no particular significance was attributed to these observations. However, the analysis presented here shows a common pattern of interactions is present in the coordination of intra-subunit K^+^ ions by clusters of carbonyl groups occurs in all of the still limited number of halophilic proteins crystallised in the presence of potassium ions. Given the related architecture seen in the K^+^ binding sites in D2HDH, GlcDH and MSH and the intra-subunit K^+^ sites in MDH and ferredoxin, where the ligands are clustered in depressions on the protein surface and formed mainly by carbonyls, equivalent sites are likely to be found more widely in other halophilic proteins. This apparent generic use of carbonyl clusters to coordinate K^+^ ions suggests that interactions between them may contribute to the adaptation of the protein to its high salt environment, possibly by stabilisation of its folded state, in a manner that is completely independent of, and additional to, any interactions involving the carboxyl groups of the many acidic surface residues. However, the relative contribution to halophilicity and precise role of these carbonyl-K^+^ ion interactions compared to other stabilising effects is yet to be determined and, as the predominant coordinating groups are main chain carbonyls, testing the contribution of these sites to halophilic adaptation by mutagenesis is not straightforward.

## Materials and methods

### Protein expression and purification

Sulfur and seleno-methionine recombinant D2HDH were expressed in *E.coli* in an insoluble form. Cells were disrupted by sonication and the enzyme extracted from the cell debris in 8 M urea in buffer A (20 mM Tris pH 8.0, 2 mM EDTA) with 50 mM DTT, and refolded by rapid 10-fold dilution into a solution of 4 M NaCl in buffer A^27,53^. For purification, ammonium sulfate was added to the refolded protein solution to 3.7 M, the pellet separated by centrifugation and the supernatant applied to a DEAE sepharose or Ether-Toyopearl (TOSOH) column pre-equilibrated in 3.8 M ammonium sulfate in buffer A, followed by elution with 4 M NaCl in buffer A. Fractions with eluted protein were collected and applied to a 1.6 × 60 cm HiLoadSuperdex200 gel-filtration column equilibrated with 2 M NaCl or 1 M KCl in buffer A. Peak fractions containing D2HDH were combined and concentrated prior to crystallization to 10-12 mg ml^−1^ (extinction coefficient calculated as 28545 M^−1^ cm^−1^) in buffer A with 1 M NaCl or 1 M KCl.

### Crystallization and structure determination

#### (a) crystallisation of D2HDH with NAD^+^, 2-ketohexanoic acid and NaCl - pdb:8qzb

Preliminary crystallisation trials of D2HDH in the presence of 5 mM NAD^+^ and 50 mM 2-ketohexanoic acid, conditions that could lead to the formation of a non-productive ternary complex, yielded two distinct crystal forms (forms I and II)^53^. Crystals of sulfur and seleno-Met D2HDH in form II grew using 1:1 sitting drops of protein and precipitant solutions of 0.1 M Tris-HCl pH8, 0.5 M magnesium acetate and 20% PEG3350 and diffracted to high resolution^53^. A single Se-Met form II crystal was cryo protected in crystallisation buffer containing 25% ethylene glycol and three wavelength MAD data were collected to 2.0 Å at 100 K using beamline I02 of the Diamond synchrotron and processed in space group P1 using Xia2^54^ (Supplementary Table 5). The selenium substructure and preliminary map were calculated using SHELXC/D/E^55^. A substantially complete model for the four D2HDH chains in the asymmetric unit was built and refined semi-automatically, using the CCP4 suite of programs^56^. Subsequently, data from a single sulfur-Met D2HDH crystal grown in the same conditions and in the same space group were collected at the Diamond Light Source (DLS) on station I03 at a wavelength of 0.9801 Å and temperature of 100 K, processed to 1.16 Å and the structure determined using molecular replacement with the Se-Met D2HDH structure as the search model. Following rounds of rebuilding and refinement using Coot^57^ and Refmac^58^, a final model was produced (Table 1). The electron density was in general of very high quality (Supplementary Fig. 2b) enabling the chain to be traced for all residues, the only exceptions being some partial disorder of a small number of residues at the chain termini in the different subunits, with 98.3 and 1.7% of the residues in the favoured and allowed regions of the Ramachandran plot, respectively. The asymmetric unit contained two D2HDH dimers (AB and CD), related by non-crystallographic translational symmetry (0, 0.5, 0.5). Although the position of domain d2 of subunit D was clear, the fit of this domain to the density was not as good as the rest of the structure indicating some static disorder and this is reflected in the lower than expected overall RSRZ value. Additional density corresponding to the NAD^+^ co-factor and the 2-ketohexanoic acid could also be identified. In the structure of this crystal form the four subunits are arranged as two independent dimers, with the expected fold and quaternary structure for the 2HADH family^29,59,60^, but with small differences in the relative domain orientation in the different subunits. The structure contained 18 Mg^2+^, 8 Na^+^, 4 Cl^-^ and 1622 waters together with a small number of buffer components, with all being identified by a combination of electron density, ligand bond lengths and geometry^61^. The waters were assigned to the four subunits using Watertidy^56^ together with visual inspection, identifying 400, 470, 403, and 349 water molecules for chains A, B, C, and D, respectively. Those waters in equivalent positions in all four subunits and separately in chains A, B, and C (defined as being within 1.0 Å of each other) were identified by superposition of the individual domains of each subunit to allow for differences in domain orientation.

#### (b) crystallisation of D2HDH with NAD^+^, 2-ketohexanoic acid, SO_4_^2-^ and NaCl - pdb:5mh6

Data were also collected to 1.35 Å resolution on DLS station I02 at 100 K and a wavelength of 0.97204 Å on crystals of a similar complex of D2HDH, but where the final gel filtration step of the purification had been omitted, leaving residual sulfate in the crystallisation mixture. The crystals were in space group P1 with two dimers in the asymmetric unit and the structure determined using molecular replacement and refined as before (Table 1). The final model had 98.2 and 1.8% of the residues in the favoured and allowed regions of the Ramachandran plot, respectively.

#### (c) crystallisation of D2HDH in KCl and identification of counter ions - pdb:9ibe

Crystals of D2HDH in the presence of 5 mM NADP^+^ and 50 mM 2-ketohexanoic acid were also grown in 0.1 M Tris-HCl pH8, 0.1 M magnesium acetate and 26% PEG3350, but with the protein prepared in 1 M KCl. The crystals were also in space group P1, with two dimers in the asymmetric unit. Data were collected on station I03 at a wavelength of 0.940535 Å and temperature of 100 K, to 1.26 Å resolution and the structure determined by molecular replacement using the coordinates of the individual domains of 8qzb as the search model and refined using Refmac5, with 95.7 and 3.3% of the residues of the final model in the favoured and allowed regions of the Ramachandran plot, respectively (Table 1). To verify the positions of potential K^+^ and Cl^-^ ions in the structure, data were also collected from crystals grown in the same conditions on station I23 of the Diamond synchrotron at energies above and below the absorption edges of these two ions (5.0 and 3.5 KeV for K^+^; 3.5 and 2.75 KeV for Cl^-^). For completeness, data were also collected at 3.9 KeV to exclude the remote possibility that Ca^2+^ was contributing to the anomalous signal, despite not being present in the crystallization conditions. The anomalous substructure was calculated using ShelX/Anode^55,62^, with peaks above 5 sigma in the 5 KeV maps taken as potential counter ions, cross-referenced with anomalous maps at the other energies and assigned consistent with the electron density, ligand bond lengths and geometry. Peaks corresponding to the protein sulfur and dinucleotide phosphorous atoms and the solvent Mg^2+^ ions were present in anomalous maps calculated at all the energies and were discounted from the K^+^ and Cl^-^ peak assignments.

#### (d) crystallisation of D2HDH with NADP^+^, 2-ketohexanoic acid and NaCl - pdb:5mh5

Crystals were also grown of NaCl prepared D2HDH in the presence of 5 mM NADP^+^ and 50 mM 2-ketohexanoic acid in 0.1 M Tris-HCl pH8, 0.5 M magnesium acetate and 18% PEG3350. Analysis of data collected to 1.4 Å, on DLS station I03 at 100 K, at a wavelength of 0.9507 Å (Table 1) showed they belonged to a different crystal form, in space group P2_1_, with a single dimer in the asymmetric unit. The structure was therefore determined by molecular replacement using the protein coordinates of the NAD^+^/2-ketohexanoic acid complex. The electron density map was of generally high quality throughout and with clear density for both the NADP^+^ and 2-ketohexanoic acid, with the final refined model having 98.4 and 1.6% of the residues in the favoured and allowed regions of the Ramachandran plot, respectively (Supplementary Fig. 2e).

#### (e) crystallisation of D2HDH with NADPH, 2-ketohexanoic acid and NaCl - pdb:5mha

NaCl prepared D2HDH was further crystallised, but in the presence of a mixture of 5 mM NADPH and 50 mM 2-ketohexanoic acid in 0.1 M Tris-HCl pH8, 0.5 M magnesium acetate and 24% PEG3350. The resultant crystals were also in space group P2_1_ but with a significant change to the b axis cell dimension compared to the NADP^+^/2-ketoacid complex (pdb:5mh5) (Table 1). Data were collected to 1.6 Å resolution on DLS station I04 at 100 K and a wavelength of 0.9763 Å and the structure was determined by molecular replacement as described above. This crystal form contains a dimer in the asymmetric unit with both subunits in a similar closed conformation to that of the NADP^+^/2-ketoacid complex, with the final model having 98.4 and 1.6% of the residues in the favoured and allowed regions of the Ramachandran plot, respectively. Interpretation of the electron density for the co-factor suggested that the NADPH had been oxidised by catalytic turnover during the course of crystallisation. In contrast, modelling the density for the substrate was more complicated, suggesting, in subunit B, that it represented a 2:1 mixture of the keto and hydroxy acids and the final model incorporates both these molecules (Supplementary Fig. 2c, 3). In subunit A refinement indicated that the proportion of the hydroxy acid was significantly lower, and thus the final model contained coordinates for the ketoacid alone in this subunit.

When a 2-ketohexanoic acid in the abortive orientation (with its *re* face packed against the nictotinamide ring) was fitted to the density for the B subunit and refined, inspection of the resulting map showed a clear 6 sigma difference feature out of the plane of the ketoacid and distal to the nicotinamide ring some 1.5 Å from its C2 atom (Supplementary Fig. 4a). The height and position of this difference peak suggests that it arises from the presence of an oxygen atom covalently bound to C2 and is best explained as representing the hydroxyl of D-2-hydroxyhexanoic acid bound in the productive orientation. However, refining the structure with just this D-2-hydroxyhexanoic acid present instead of the ketoacid, resulted in a 12 sigma difference feature at the position of the C2 hydrogen (Supplementary Fig. 4b). These data implied that the active site in this subunit contains a mixture of both of these substrates and further modelling and refinement suggested an approximately 2:1 ratio of 2-ketohexanoic acid and D-2-hydroxyhexanoic acid in the abortive and productive orientations, respectively (Supplementary Fig. 4c,d,e).

#### (f) crystallisation of D2HDH in the apo form - pdb:8qza

Crystals of D2HDH were also grown from NaCl prepared protein in 0.1 M Tris-HCl pH8, 0.5 M magnesium acetate and 20% PEG3350, but without addition of cofactor or substrate. These crystals grew in space group P2_1_ with a dimer in the asymmetric unit, but again with significant differences in the b and c cell dimensions compared to the complex structures. Data were collected to 2.25 Å resolution on DLS station I03 at 100k and a wavelength of 0.9801 Å and the structure determined by molecular replacement (Table 1). In the refined structure both d2 domains of the dimer and the d1 domain from subunit B were well defined, but the density for the d1 domain in the A subunit was much weaker, indicating some static disorder in the position of this domain. The final model had 96.4 and 3.6% of the residues in the favoured and allowed regions of the Ramachandran plot, respectively.

### Ultracentrifugation

Samples of D2-HDH were used for analytical ultracentrifugation experiments to elucidate the oligomeric state of the protein using a XL-A ultracentrifuge. Different conditions were assayed varying NaCl concentration (1 and 4 M) and protein concentration (0.5 and 1 mg/ml). Samples were run at 40,000 rpm at 20.0 °C. The experiment was recorded using a 12 mm optical path length cell and UV absorption optics set at 280 nm and analysed using the method of Schuck et al.^63^ The sedimentation coefficients were normalised to standard solvent conditions of the density and viscosity of water at 20.0 °C. The assignment of each oligomeric species to a particular sedimentation coefficient was based on a dimer molecular weight of ~66 kDa and aspect ratio (hydrodynamically equivalent prolate ellipsoid) of ~2:1, taking into consideration hydrodynamic non-ideality and protein solvation.

### Enzyme assays

The activity of D2HDH was determined at room temperature (22 °C) at pH 8.0 (50 mM HEPES) by following the oxidation of NAD(P)H by measuring the decrease in absorbance at 340 nm over 3 min, using enzyme concentrations of 0.35 0.45 µM (Supplementary Data 2). For measurements in triplicate of activity under conditions of varying KCl concentrations (50 mM-4 M) and also under varying concentrations of MgCl_2_ (0–1.5 M, in duplicate for NADH) in the presence of 1 M KCl, reaction mixtures contained 0.3 mM cofactor and 4 or 20 mM 2-ketohexanoic acid for the assays with NADPH and NADH, respectively. Assays (in duplicate) for determining kinetic parameters of D2HDH were performed in the same manner, but using 0.25 mM cofactor.

The dependence of D2HDH activity in the presence of organic solvents (20% concentrations of DMSO, glycerol or ethanol) was measured in triplicate under varying concentrations of 2-ketohexanoic acid at 40 °C in mixtures containing 2 M NaCl, Tris-HCl pH 8.0, 0.3 mM NADH.

Thermofluor based assays^64^ were used to determine the melting temperature of D2HDH under varying concentrations of KCl (50 mM-4 M), NaCl (50 mM-4 M) and MgCl_2_ (0–0.5 M in 3 M KCl). 50 µl assay mixtures of 0.6 mg/ml D2HDH, 50 mM HEPES pH 8.0, 0.1 µl SYPRO orange solution (SIGMA) and the required salt were prepared in triplicate on a 96 well plate and fluorescence measured on a Stratagene MX3005P RTPCR machine running MXPro software as over a range of 25–98 °C. Melting temperatures (T_m_) were calculated from the data using excel.

### Structure comparisons

To facilitate the identification of features relating to halophilic adaptation three comparison data sets were compiled using structures taken from the RCSB (access date 1-12-21). The first of these, the MESO2HADH comparison set, comprised the sequences and structures of mesophilic representatives from the eight major 2HADH subfamilies excluding the DDH subfamily^29^ (Supplementary Table 2). The second reference data set, the SALTIN comparison set, was comprised of eight proteins from other salt-in halophiles whose structures have been determined at a resolution greater than 1.6 A (Supplementary Table 2). The third data set, the MESOSALTIN comparison set, was comprised of mesophilic homologues of proteins in the SALTIN set (Supplementary Table 2, Supplementary Data 1). For each protein, solvent and substrate atoms, as well as any residues in an expression tag, were removed prior to analysis. The solvent-accessible surface areas for each atom of these structures were calculated using AREAIMOL^65^. The definitions of Miller et al.^66^ for nonpolar, polar, and charged constituents of proteins were used to tabulate the chemical composition of the surface.

### Statistics and reproducibility

Where XY line graphs were used to represent the biochemical data, all data points are shown with the number of replicates given in the legends. Statistical analysis for the data processing of the crystallographic data shown in Table 1 used the algorithms of the processing software detailed in methods.

### Reporting summary

Further information on research design is available in the Nature Portfolio Reporting Summary linked to this article.

## Supplementary information

Supplementary Information

Supplementary Data 1

Supplementary Data 2

Description of Additional Supplementary Files

Reporting Summary

## Data Availability

Biological samples supporting the findings of this manuscript are available from the corresponding authors upon reasonable request. Atomic coordinates for D2HDH with NAD^+^, 2-ketohexanoic acid and NaCl (pdb:8qzb), D2HDH with NADP^+^, 2-ketohexanoic acid and KCl (pdb:9ibe), D2HDH with NADP^+^, 2-ketohexanoic acid and NaCl (pdb:5mh5), D2HDH with NADPH, 2-ketohexanoic acid, 2-hydroxyhexanoic acid and NaCl (pdb:5mha), D2HDH with NAD^+^, 2-ketohexanoic acid, SO_4_^2-^ and NaCl (pdb:5mh6) and D2HDH in the apo form (pdb:8qza) have been deposited in the RCSB Protein Data Bank. Numerical values for Fig. 4 can be found in Supplementary Data 2.
