# Supplementary material for: Potassium binding by carbonyl clusters, halophilic adaptation and catalysis of Haloferax mediterranei D-2-hydroxyacid dehydrogenase
Source: Commun Biol. 2025 Aug 6;8:1170. doi: 10.1038/s42003-025-08587-7 (PMC12328707; doi:10.1038/s42003-025-08587-7)
Supplement: Supplementary file 1 — Supplementary Information [file 42003_2025_8587_MOESM1_ESM.pdf]

Supplementary Information

Potassium binding by carbonyl clusters, halophilic adaptation and catalysis of *Haloferax mediterranei* D-2-hydroxyacid dehydrogenase

Jessica Domenech, Nuttawan Pramanpol, Claudine Bisson, Sveta E. Sedelnikova, Joshua R. Barrett, Abdul A. A. B. Dakhil, Ali S. Abdelhameed, Stephen E. Harding, David W. Rice, Patrick J. Baker and Juan Ferrer

Supplementary Fig. 1. Quaternary Structure of D2HDH

Supplementary Fig. 2. Representative electron density maps

Supplementary Fig. 3 Proposed reaction mechanism for D2HDH

Supplementary Fig. 4 The substrate binding site of the D2HDH/NADP^{+}/2-ketohexanoic acid/D-2-hydroxyhexanoic acid complex

Supplementary Fig. 5 K^{+} and water binding to the acidic surface of the D2HDH dimer

Supplementary Fig. 6 The similarity between K^{+} and Na^{+} binding sites in D2HDH

Supplementary Fig. 7 Mg^{2+} and Cl^{-} binding to the D2HDH/KCl/NADP^{+}/2-ketohexanoic complex

Supplementary Table 1 Relative domain closure of the individual chains in the D2HDH cofactor/substrate complex structures compared to the position of domains d1 and d2 in the B subunit of the apo structure.

Supplementary Table 2a Percentage of residue types in the primary sequence of representative members of the 2HADH family

Supplementary Table 2b Percentage of exposed surface area by atom type and for side chains of selected residues in the dimer of *H. mediterranei* D2HDH compared to the biological assemblies of proteins in the comparison data sets.

Supplementary Table 3 Average temperature factors for the two domains of each subunit of the two dimers in the 1.16 Å structure of the D2HDH/NAD^{+}/2-ketohexanoic acid complex

Supplementary Table 4 Location and ligands for the 7 potassium ion sites in the D2HDH/KCl/NADP^{+}/2-ketohexanoic acid structure.

Supplementary Table 5 X-ray data collection statistics for D2HDH Se-Met

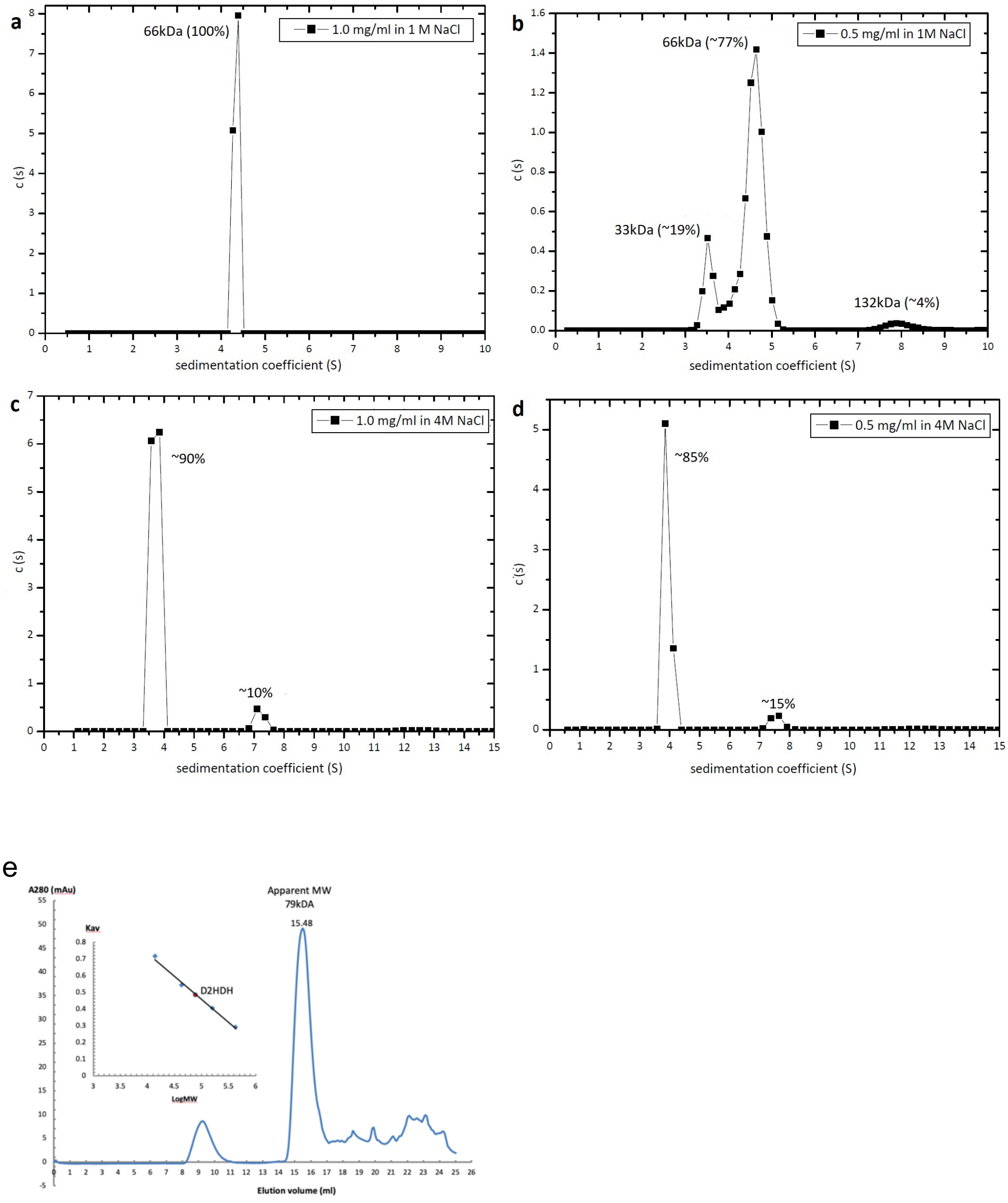

Supplementary Fig. 1. Quaternary Structure of D2HDH

Ultracentrifugation experiments on D2HDH, showing the presence of (a) the dimer (~4.7S) at low salt concentration at a protein concentration of 1.0 mg/mL; (b) a mixture of monomeric, dimeric (~3.5S) and tetrameric (~7.8S) species with diluted protein (0.5 mg/mL) at low salt concentration and (c, d) a mixture of dimeric and tetrameric species in high salt. The lower apparent values for the dimer (~3.8S at 1mg/mL and ~4.0S at 0.5mg/mL) in 4M NaCl compared to ~4.7S in 1 M NaCl are likely to be due to higher hydrodynamic non-ideality or different hydrations at the high salt concentrations, and/or due to the presence of some unresolved monomer species. (e) Gel filtration performed on a Superdex200 Increase column equilibrated in 4M KCl 50mM tris-HCl buffer at a flow rate of 0.5ml/min, with D2HDH eluting as a main peak of 14.48 ml. Insert shows the calibration plot for the gel filtration column obtained under the same conditions, with Ferritin (440 kDa), Aldolase (158 kDa), Ovalbumin(43 kDa), and Ribonuclease (13.7 kDa), shown as blue squares. D2HDH (red circle) has a Kav=0.48, which corresponds to an apparent MW of 79kDa.

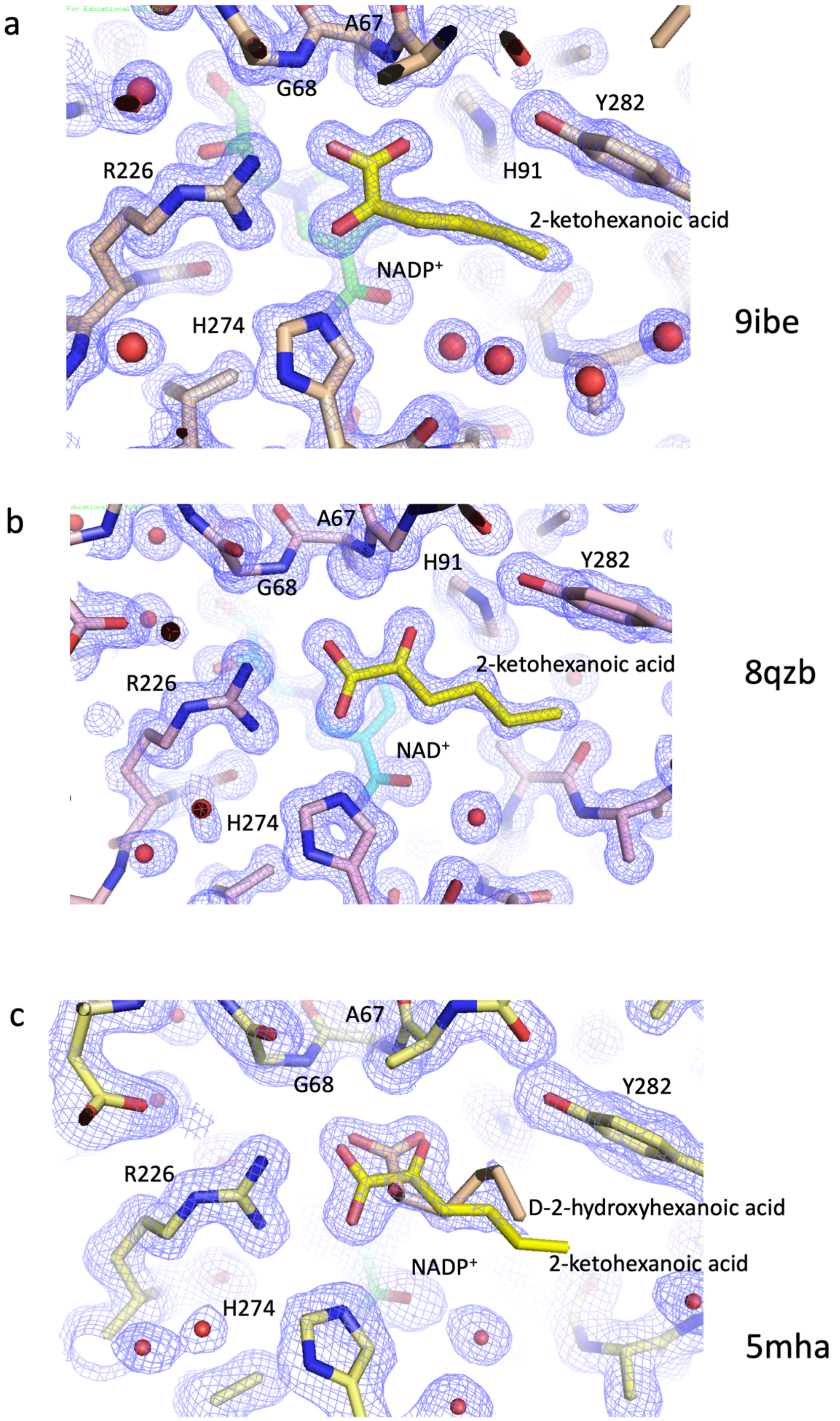
a
A67
G68
Y282
R226
H91
2-ketohexanoic acid
NADP+
H274
9ibe
b
A67
H91
Y282
G68
2-ketohexanoic acid
R226
8qzb
NAD+
H274
c
A67
G68
Y282
R226
D-2-hydroxyhexanoic acid
2-ketohexanoic acid
NADP+
H274
5mha

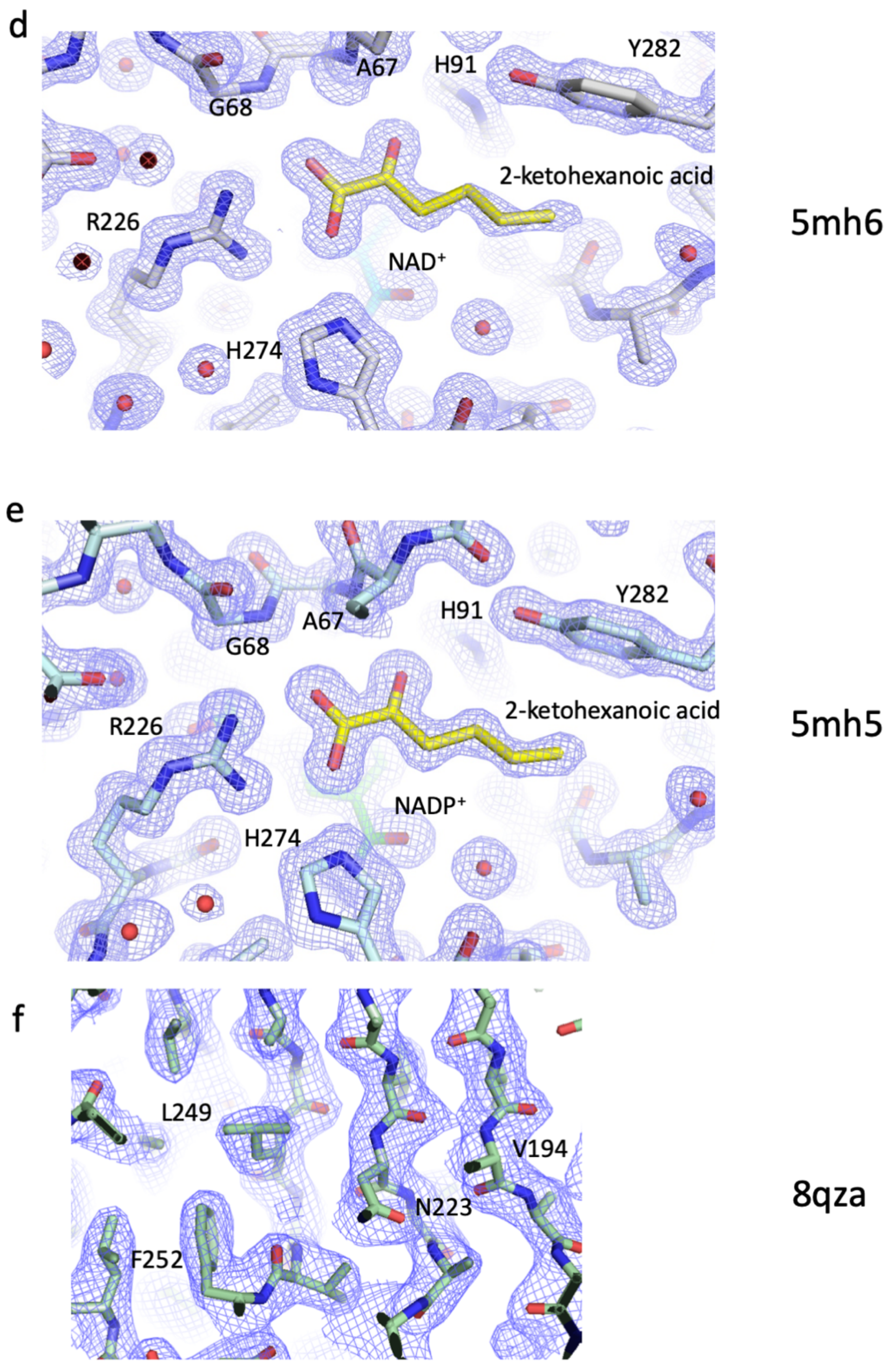

Supplementary Fig. 2. Representative electron density maps

2Fo-Fc electron density maps for the (a) 1.26 Å D2HDH/ KCl/NADP$^+$/2-ketohexanoic acid complex (contoured at 1.4σ, pdb:9ibe), (b) 1.16 Å D2HDH/NaCl/NAD$^+$/2-ketohexanoic acid complex (contoured at 1σ, pdb:8qzb) (c) 1.57 Å D2HDH/ NaCl/NADP$^+$/2-ketohexanoic acid/D-2-hydroxyhexanoic acid complex (contoured at 0.8σ, pdb:5mha) (d) 1.35 Å D2HDH/NaCl/NADP$^+$/2-ketohexanoic acid complex (contoured at 1σ, pdb:5mh6) (e) 1.4 Å D2HDH/ NaCl/NAD$^+$/2-ketohexanoic acid complex (contoured at 1σ, pdb:5mh5) and (f) the 2.25 Å apo D2HDH structure (contoured at 1σ, pdb:8qza).

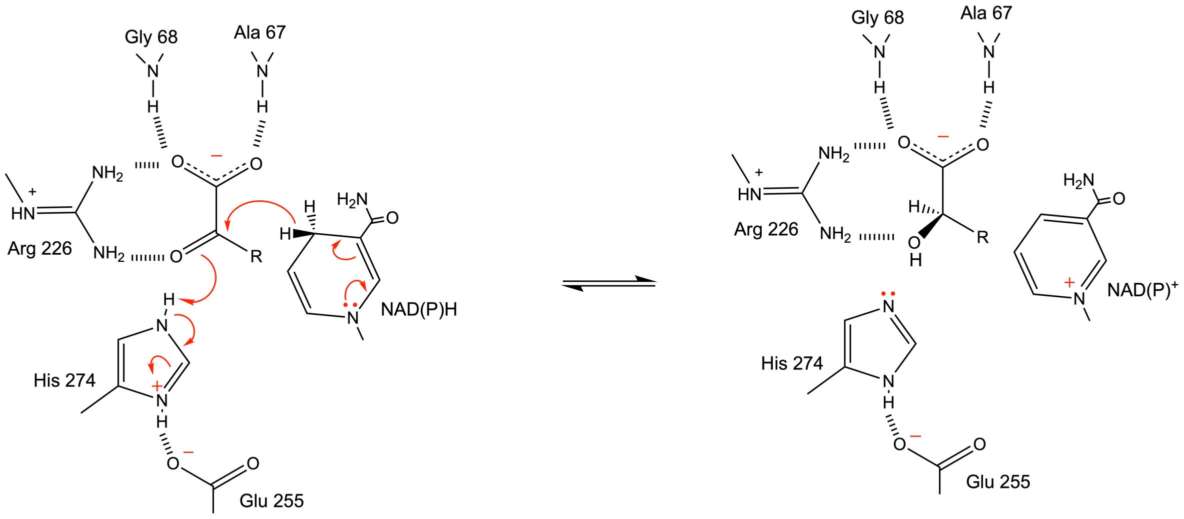

Supplementary Fig. 3 Proposed reaction mechanism for D2HDH. Curly arrows show the flow of electrons, with the NAD(P)H C4 hydride attacking the C2 of the 2-ketoacid, stabilised by Arg 226 and the main chain NH of Ala 67 and Gly 68, with His 274 providing the acid base catalysis.

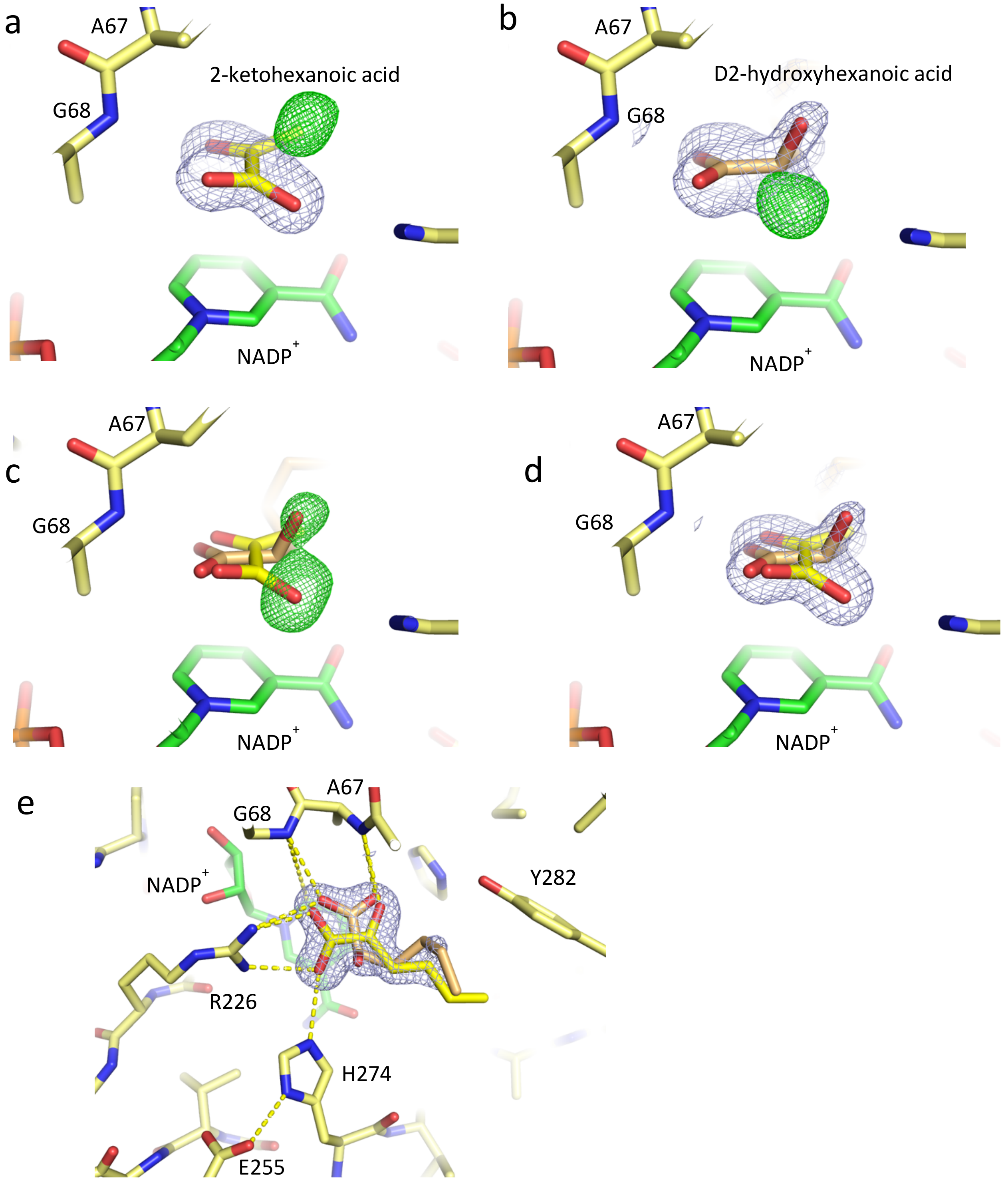

Supplementary Fig. 4 The substrate binding site of the D2HDH/NADP$^+$/2-ketohexanoic acid/D-2-hydroxyhexanoic acid complex (pdb:5mha), *2Fo-Fc* maps in blue, positive *Fo-Fc* maps in green and the NADP$^+$ with green carbons. (a) refinement with 2-ketohexanoic acid alone (yellow carbons) showing a positive 6σ difference feature, (b) refinement with D-2-hydroxyhexanoic acid alone (brown carbons) showing a positive 12σ difference feature, (c) the 2-ketohexanoic acid and D2-hydroxyhexanoic acid of the final refined model, showing that the difference features in (a) and (b) correspond to the positions of the C2-hydroxyl in the productive orientation and C2-keto oxygen in the abortive orientation, respectively. (d) The *2Fo-Fc* map for the substrate of the final model, with a mixture of 2-ketohexanoic acid (occupancy 0.67) and D-2-hydroxyhexanoic acid (occupancy 0.33), (e) a zoomed out view, showing the less clear density for the aliphatic tail of the substrate.

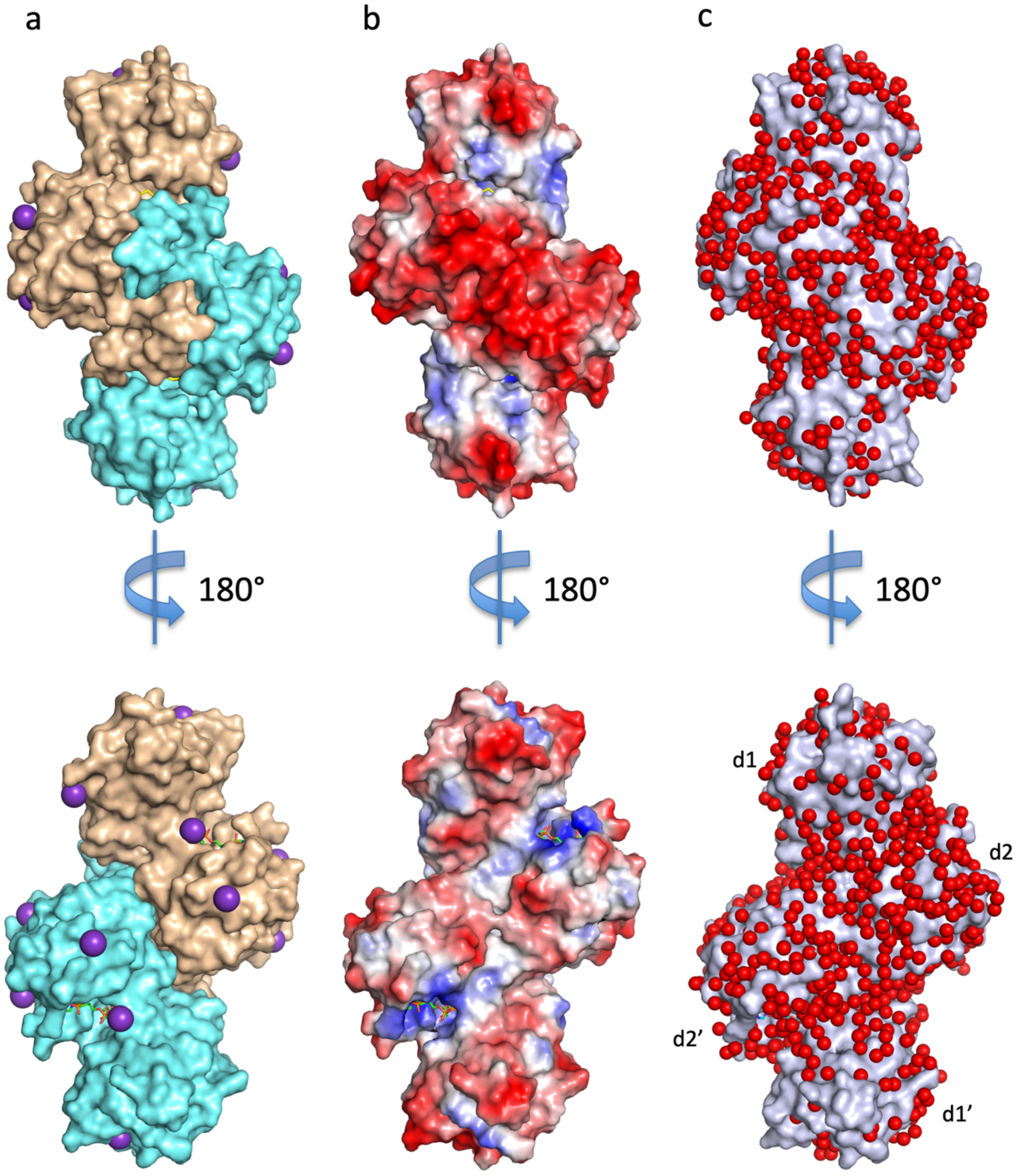

Supplementary Fig. 5 K^+ and water binding to the acidic surface of the D2HDH dimer. Two views of the dimer, related by 180º, showing: (a) binding of K^+ (purple spheres) to the D2HDH/$NADP^+$/2-ketohexanoic acid complex (brown/cyan, pdb:9ibe); (b) the D2HDH dimer electrostatic surface, highlighting large areas of negative charge prepared using Pymol and default colours (red negative, blue positive); (c) the surface of the dimer of the D2HDH/NAD^+/2-ketohexanoic acid complex (grey, pdb:8qzb) showing water (red spheres). In each case substrate and $NAD(P)^+$ are shown in stick format.

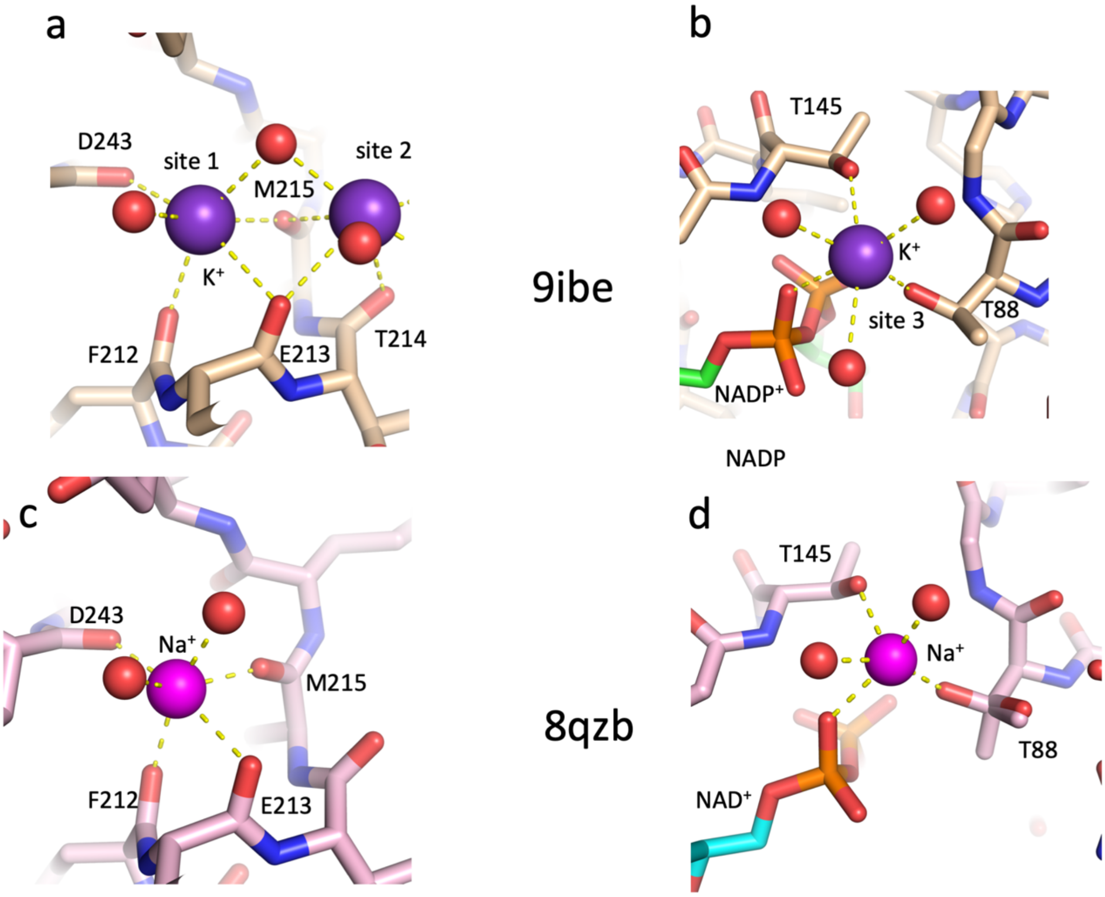

Supplementary Fig. 6. The similarity between K^+ and Na^+ binding sites in D2HDH. K^+ ion binding to (a) site 1 and (b) site 3 in the D2HDH/KCl/NADP$^+$/2-ketohexanoic complex (pdb:9ibe) and the equivalent regions in the D2HDH/NaCl/NAD$^+$/2-ketohexanoic complex (pdb:8qzb), showing Na^+ binding (c) in site 1 and (d) site 3.

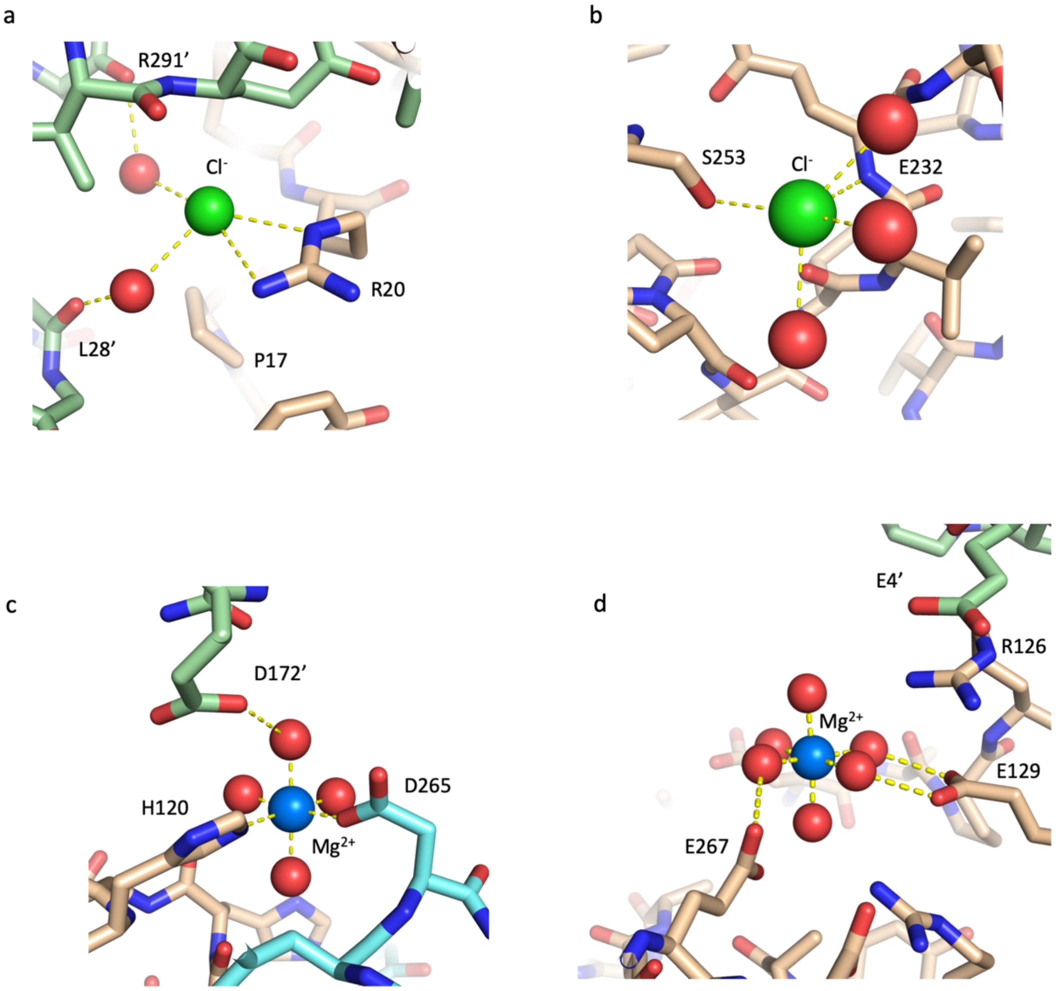

Supplementary Fig. 7 Mg^{2+} and Cl^- binding to the D2HDH/KCl/$NADP^+$/2-ketohexanoic complex (pdb:9ibe). Two separate chloride ion binding sites (a) the site in region of crystal contacts and (b) the inter-subunit site. (c) Mg^{2+} binding between two subunits (beige and light blue) of the D2HDH dimer, and (d) Mg^{2+} binding between two symmetry related subunits in a region of crystal contacts. In each panel crystal symmetry related molecules are shown with light green carbons, Cl^- , Mg^{2+} and waters as green, blue and red spheres, respectively. For clarity only those water molecules binding directly to the ions are shown.

Supplementary Table 1				
D2HDH Structure	PDB Code	Asymmetric Unit contents	Chain	Relative domain closure
Apo-enzyme	8qza	1 dimer	A	#
			B	0°
$NADP^+$/2-ketohexanoic acid (KCl)	9ibe	2 dimers (AB and CD)	A	13.4°
			B	17.4°
			C	15.8°
			D	17.8°
NAD^+/2-ketohexanoic acid	8qzb	2 dimers (AB and CD)	A	13.4°
			B	16.7°
			C	15.0°
			D	14.7°
$NADP^+$/2-ketohexanoic acid	5mh5	1 dimer	A	16.3°
			B	15.1°
$NADP^+$/2-ketohexanoic acid/2-hydroxyhexanoic acid	5mha	1 dimer	A	17.7°
			B	15.6°
NAD^+/2-ketohexanoic acid/SO_4^{2-}	5mh6	2 dimers (AB and CD)	A	16.6°
			B	13.5°
			C	14.6°
			D	17.3°

Supplementary Table1. Relative domain closure of the individual chains in the D2HDH cofactor/substrate complex structures compared to the position of domains d1 and d2 in the B subunit of the apo structure.
A value for the domain closure of the A subunit of the apo structure is not included due to weak density for its d1 domain.

Supplementary Table 2a Percentage of residue types in the primary sequence of representative members of the 2HADH family compared to the sequence of D-2-hydroxyacid dehydrogeanse from *Haloferax meditteranei*, calculated from UniprotKB sequences

**PDB Code**	**5mha**			**3ba1**	**5tx7**	**1j4a**	**4cuj**	**2o4c**	**3oet**	**4xcv**	**4zqb**	**3jtm**	**4xyb**	**1mx3**	**1hku**	**4njm**	**2g76**	**4lsw**
**Clade**	**DDH**	**2HADH average**	**sd**	**GHRB**	**GHRC**	**LDHD**	**LDHD**	**PDXB**	**PDXB**	**GHRA**	**GHRA**	**FDH**	**FDH**	**CTBP**	**CTBP**	**SERA**	**SERA**	**GHRB**
**Asp (%)**	**8.8**	**5.8**	1.2	5.8	6.1	8.7	5.8	6.8	6.3	6.9	4.8	5.5	5.1	5.2	5.3	4.3	3.8	7.2
**Glu (%)**	**9.4**	**6.9**	1.4	7.7	4.6	7.5	6.7	6.1	6.9	7.5	8	6.5	6.9	5.9	6	11	5.8	6
**Lys (%)**	**0.6**	**4.3**	2.9	5.4	1.8	6.6	5.5	0.8	3.4	3.8	1.3	7	4.9	3.2	3	12	4.9	1.6
**Arg (%)**	**6.5**	**6.4**	1.9	5.8	7.7	4.2	4.3	10.3	7.4	7.8	8.6	4.9	5.4	6.4	6.5	4.3	4.1	8.2
**His (%)**	**3.2**	**2.5**	1.1	1.3	2.8	2.4	2.7	1.6	2.6	1.9	3.2	2.3	3.8	4.5	4.4	0.7	1.5	1.9
**Gln (%)**	**1**	**2.7**	1.1	1.3	1.5	3.3	4	3.2	1.9	2.5	1.3	3.4	1.3	3.4	3.5	2	5.1	2.2
**Asn (%)**	**1.6**	**3.4**	1.1	3.2	3.7	4.2	5.2	1.6	3.2	2.5	1	4.7	3.8	3.4	3.3	4.7	3.6	2.5
**Ser (%)**	**4.2**	**4.4**	1.3	5.1	5.8	2.1	4.6	3.7	3.4	4.4	3.8	4.4	5.4	5.5	5.6	1.7	6	4.1
**Thr (%)**	**6.2**	**5.1**	0.7	4.8	6.4	4.8	5.5	4.2	5.6	5	4.5	4.9	4.3	4.8	5.6	4.7	6.6	4.7
**Tyr (%)**	**2.6**	**2.3**	1	2.2	1.8	3.6	3	2.1	2.4	1.3	1.9	3.9	4.1	1.6	1.6	2.3	0.6	1.9
**Cys (%)**	**0.6**	**1.4**	0.7	1.9	1.2	0	1.8	2.1	1.3	0.3	1	1.3	1.8	1.8	1.9	2.3	2.4	0.6
**Ala (%)**	**12**	**10.8**	2.4	7.7	12	10.2	10.3	14.2	10.8	8.8	15	9.6	11	11.4	11.2	6	10.1	14.2
**Phe (%)**	**3.9**	**3.3**	1.2	3.8	2.5	3.3	5.8	1.8	3.2	5.3	1.9	2.9	2.3	2.5	2.6	4.7	2.8	3.8
**Gly (%)**	**8.4**	**8.3**	0.9	8.6	8.3	6.6	7	9.2	8.2	8.8	8	8.6	8.4	8	7.4	9.7	9.4	8.8
**Ile (%)**	**3.2**	**5.7**	2.1	5.8	4.3	6.6	4.9	3.2	4.2	5.6	4.8	5.7	5.9	5.5	5.8	12.7	4.5	5.7
**Leu (%)**	**7.8**	**10.3**	2.5	11.5	11.3	6.6	11.6	12.6	13.2	9.7	12.5	8.6	9	10.5	9.5	4	12.2	11.3
**Met (%)**	**1.9**	**2.1**	0.7	1.6	1.8	3	2.7	0.5	1.3	2.5	1.3	3.1	2.3	1.8	2.1	3	2.4	2.2
**Pro (%)**	**5.2**	**5.1**	1.4	5.1	6.4	3.9	2.4	5.3	5.6	5	7.3	4.7	6.4	6.4	6.3	2.3	4.9	5
**Val (%)**	**11.7**	**8.1**	1.4	10.9	8.3	11.4	6.4	8.9	8.2	8.8	7.7	6.8	6.6	7.7	7.7	7.4	8.3	6.9
**Trp (%)**	**1**	**1**	0.6	0.6	1.5	0.9	0	1.8	0.8	1.6	2.2	1	1.3	0.7	0.7	0	1.1	1.3

**PDB Code**	**2HADH Clade**	**Enzyme**	**Species**	**PDB Code**	**2HADH Clade**	**Enzyme**	**Species**
5mha	DDH	D-2-hydroxyacid dehydrogenase	*Haloferax mediterranei*	3jtm	FDH	Formate dehydrogenase	*Arabidopsis thaliana*
3ba1	GHRB	hydroxyphenylpyruvate reductase	*Plectranthus scutellarioides*	4xyb	FDH	Formate dehydrogenase	*Granulicella mallensis MP5ACTX8*
5tx7	GHRC	D-isomer specific 2-hydroxyacid dehydrogenase	*Desulfovibrio vulgaris*	1mx3	CTBP	CtBP dehydrogenase	*Homo sapiens*
1j4a	LDHD	D-Lactate dehydrogenase	*Lactobacillus bulgaricus*	1hku	CTBP	CtBP dehydrogenase	*Rattus norvegicus*
4cuj	LDHD	D-Lactate dehydrogenase	*Salmonella enterica*	4njm	SERA	3-phosphoglycerate dehydrogenase	*Entamoeba histolytica*
2o4c	PDXB	D-Erythronate-4-phosphate dehydrogenase	*Pseudomonas aeruginosa*	2g76	SERA	3-phosphoglycerate dehydrogenase	*Homo sapiens*
3oet	PDXB	D-Erythronate-4-phosphate dehydrogenase	*Salmonella typhimurium*	4lsw	GHRB	2-Hydroxyacid dehydrogenase	*Ketogulonicigenium vulgare*
4xcv	GHRA	Probable 2-hydroxyacid dehydrogenase	*Rhizobium etli CFN 42*				
4zqb	GHRA	NADP-dependent dehydrogenase	*Cereibacter sphaeroides 2.4.1*				

Supplementary Table 2b	D2HDH	MESO2HADH		SALTIN		MESOSALTIN	
**Surface area atom type**	%	%	sd	%	sd	%	sd
Non-polar	43.2	49.9	2.1	43.2	2.3	50.6	3.7
Polar	56.8	50.1	2.1	56.8	2.3	49.4	3.7
Basic	8.0	10.9	1.9	8.7	2.6	9.7	3.8
Acidic	23.8	12.9	1.7	23.0	3.3	12.3	2.8
Polar uncharged	24.9	26.3	2.3	25.2	2.1	27.3	2.6
**Surface area selected residue side chains**							
Asp	14.4	7.5	1.8	17.2	3.2	7.3	2.8
Glu	19.2	12.1	2.8	14.5	4.4	11.1	3.3
Arg	11.2	11.9	4.4	9.7	4.1	8.7	5.0
Lys	1.2	9.5	6.3	5.0	2.7	11.0	4.9
Lys alkyl side chain component	0.6	5.9	3.9	2.6	1.6	6.7	3.0
FIVL non-polar side chain component	6.1	9.2	1.9	7.5	0.8	11.4	2.3

Supplementary Table 2b. Percentage of exposed surface area by atom type and for side chains of selected residues in the dimer of *H. meditteranei* D2HDH compared to the biological assemblies of proteins in the comparison data sets. Basic = side chain nitrogens from Arg, Lys and N-terminus; Acidic = carboxylate oxygens of Asp, Glu and C-terminus; FIVL = side chains of Phe, Ile, Val and Leu; side chains include CA; sd values shown are calculated for the data set means. Data sets: MESO2HADH, representatives of 2HADH family; SALTIN, representative high resolution saltin halophilic protein structures; MESOSALTIN, representative mesophilic homologues of the SALTIN data set. The proteins included in each data set are identified in Supplementary Table 2 and Supplementary Data 1.

**Supplementary Table 3**	D2HDH/NAD$^+$/2-ketohexanoic acid. pdb:8qzb		
	Average B factors		
Chain	Main Chain	Side Chain	All
A domain 1	21.1	26.4	23.7
B domain 1	15.8	20.7	18.1
C domain 1	20.6	25.0	22.8
D domain 1	28.2	33.1	30.6
Average domain 1	21.4	26.3	23.8
A domain 2	9.8	13.4	11.5
B domain 2	9.4	12.3	10.7
C domain 2	9.5	12.4	10.9
D domain 2	10.5	13.7	12.0
Average domain 2	9.8	12.9	11.3

Supplementary Table 3. Average temperature factors for the two domains of each subunit of the two dimers in the 1.16 Å structure of the D2HDH/NAD$^+$/2-ketohexanoic acid complex (pdb 8qzb)

**Supplementary Table 4**									
K^+ Site	Ligands (distance/Å) for K^+ ions in subunit A								Comments
1	F212 C=O (2.68)	E213 C=O (2.90)	M215 C=O (2.59)	D243 C=O (2.65)	HOH O (2.73)	HOH O (2.82)			
2	E213 C=O (2.89)	T214 C=O (2.89)	M215 C=O (2.94)	HOH O (2.43)	HOH O (2.98)	HOH O (2.66)	HOH O (3.26)		
3	T88 OG1 (2.69)	T145 OG1 (2.72)	NADP O1A (2.67)	HOH O (2.71)	HOH O (2.93)				
4	L57 C=O (2.75)	A59 C=O (2.74)	A80 C=O (2.80)	T82 OG1 (2.91)	HOH O (2.75)	HOH O (3.06)			
5	V171 C=O (2.84)	V174 C=O (2.73)	HOH O (2.81)	HOH O (2.85)	HOH O (2.85)	HOH O (2.87)	HOH O (2.69)		Site 5 not present in subunit D due to crystal contacts
6	E23 C=O (2.74)	S26 OG (2.85)	HOH O (3.22)	HOH O (3.39)					Site 6 not present in subunits B and D
7	G205 C=O (3.12)	G205' C=O (3.13)	HOH O (2.86)	HOH O (2.89)	HOH O (3.17)	HOH O (3.23)	HOH O (3.35)	HOH O (3.38)	Site links 2 Mg^{2+} ions in a crystal contact region bridging subunits A to D and B to C.

Supplementary Table 4 Location and ligands for the 7 potassium ion sites in the D2HDH/KCl/$NADP^+$/2-ketohexanoic acid structure (pdb:9ibe). This structure has two dimers (A-B and C-D) in the asymmetric unit. Ligand distances are given for the K^+ sites in subunit A.

**Supplementary Table 5**	SeMet D2HDH Data Collection		
Se-Met edge	Peak	Inflection	High energy remote
Wavelength (Å)	0.97957	0.97972	0.97204
Beamline	Diamond I02		
Resolution range (Å)	56.8-2.0 (2.11-2.0)	46.23-2.0 (2.11-2.0)	68.68-2.0 (2.11-2.0)
Space group	P1		
Unit cell (a,b,c/ Å; α,β,γ/^{O})	66.2, 75.8, 76.8; 109.2, 107.2, 96.0		
Total reflections	328929 (46793)	327726 (46698)	328995 (47677)
Unique reflections	85728 (12294)	85473 (12262)	85608 (12352)
Multiplicity	3.8 (3.8)	3.8 (3.8)	3.8 (3.9)
Completeness (%)	97.4 (95.9)	97.1 (95.6)	97.3 (96.3)
Mean I/σ (I)	20 (8.9)	18.6 (7.6)	25.9 (13.4)
R_{merge}	0.051 (0.124)	0.045 (0.149)	0.030 (0.084)
R_{pim}	0.049 (0.104)	0.042 (0.114)	0.032 (0.067)
Anomalous slope	1.98	1.49	1.73

Supplementary Table 5. X-ray data collection statistics for D2HDH Se-Met
