# Supplementary material for: Potassium binding by carbonyl clusters, halophilic adaptation and catalysis of Haloferax mediterranei D-2-hydroxyacid dehydrogenase
Source: Commun Biol. 2025 Aug 6;8:1170. doi: 10.1038/s42003-025-08587-7 (PMC12328707; doi:10.1038/s42003-025-08587-7)
Supplement: Supplementary file 4 — Description of Additional Supplementary Files [file 42003_2025_8587_MOESM4_ESM.pdf]

Description of Additional Supplementary Files

File name: Supplementary Data 1

Description: Surface properties of D2HDH and the MESO2HADH, SALTIN and MESOSALTIN data sets.

File name: Supplementary Data 2

Description: Source data behind the graphs in the paper
